# Reduced RNA adenosine-to-inosine editing in hippocampus vasculature associated with Alzheimer’s disease

**DOI:** 10.1093/braincomms/fcac238

**Published:** 2022-09-22

**Authors:** Philip S Crooke, John T Tossberg, Rachel M Heinrich, Krislyn P Porter, Thomas M Aune

**Affiliations:** Department of Mathematics, Vanderbilt University, Nashville, TN 37212, USA; Department of Medicine, Vanderbilt University Medical Center, Nashville, TN 37232, USA; Department of Medicine, Vanderbilt University Medical Center, Nashville, TN 37232, USA; Department of Medicine, Vanderbilt University Medical Center, Nashville, TN 37232, USA; Department of Medicine, Vanderbilt University Medical Center, Nashville, TN 37232, USA; Department of Pathology, Microbiology and Immunology, Vanderbilt University Medical Center, Nashville, TN 37212, USA

**Keywords:** A-to-I editing, Alzheimer’s disease, Alu double-stranded RNA, inflammation, neurodegeneration

## Abstract

Alzheimer’s disease is the most common form of dementia and recent studies identify a type 1 interferon response in Alzheimer’s disease possibly driving neuro-inflammation and other Alzheimer’s disease pathologies. Loss of adenosine-to-inosine editing of endogenous Alu RNAs results in accumulation of Alu double-stranded RNAs, activation of double-stranded RNA sensors, and induction of interferon and nuclear factor kappa B regulated genes. Here, we investigated if changes in adenosine-to-inosine editing were associated with presence of Alzheimer’s disease in total prefrontal cortex, total hippocampus, cortex vasculature and hippocampus vasculature using available RNA sequencing files. We found similar levels of Alu RNA adenosine-to-inosine editing in cortex and cortex vasculature from individuals with Alzheimer’s disease or normal cognition at the time of death and brain donation. We found modest and substantial loss of adenosine-to-inosine editing in hippocampus and hippocampus vasculature, respectively, in Alzheimer’s disease relative to normal cognition and increased expression of interferon and nuclear factor kappa B regulated genes in hippocampus. Unedited Alu RNAs as found in Alzheimer’s disease hippocampus vasculature were potent innate immune activators while edited Alu RNAs as found in normal cognition hippocampus vasculature were weak innate immune activators. Taken together, our results support a model whereby loss of Alu RNA adenosine-to-inosine editing in hippocampus results in innate immune activation that may contribute to Alzheimer’s disease pathogenesis.

## Teaser

Crooke *et al*. demonstrate extensive loss of adenosine-to-inosine (A-to-I) editing of endogenous Alu RNAs in Alzheimer’s disease hippocampus vasculature. A-to-I editing is a critical post-transcriptional process that prevents innate immune activation by endogenous Alu RNAs and unedited Alu RNAs found in Alzheimer’s disease hippocampus vasculature are potent innate immune activators.

## Graphical Abstract

**Graphical Abstract:**
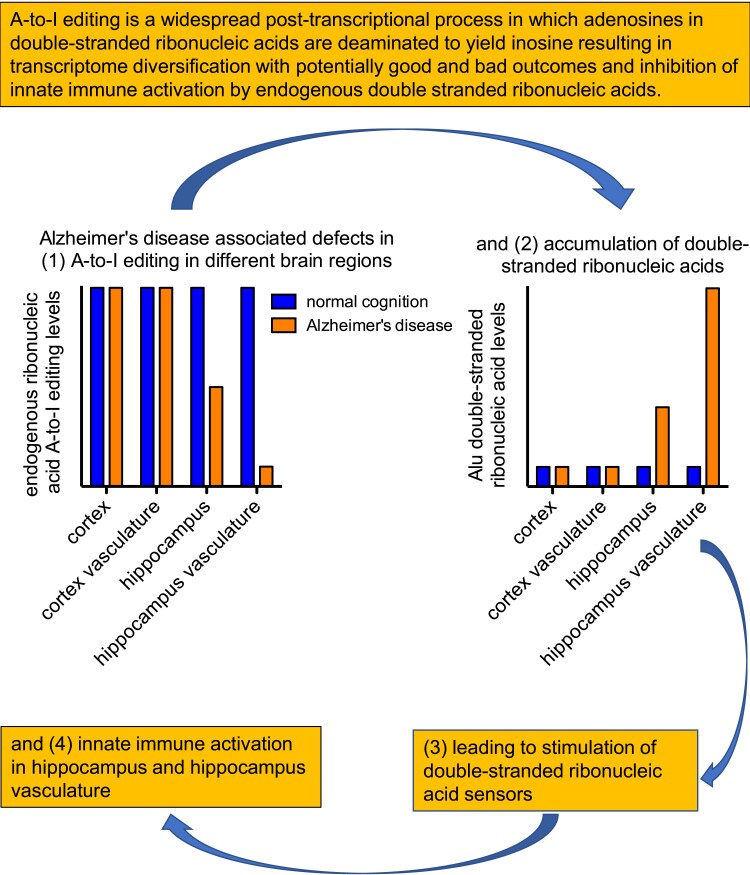

## Introduction

Deamination of adenosine-to-inosine in double-stranded RNA (dsRNA) catalyzed by the adenosine deaminase acting on RNA family of enzymes, ADAR, often referred to as A-to-I editing, is one of the most common post-transcriptional modifications in eukaryotic cells.^1–6^ The most common RNA editing sites are found in gene introns and 3′ UTRs. In contrast, editing within exons is relatively rare. The general view is that editing within introns can produce alternate splicing events and within 3′ UTRs can produce or eliminate microRNA binding sites, thus contributing to transcriptome diversification. In humans, the brain has one of the highest levels of A-to-I editing among organs suggesting that A-to-I editing is important for both brain development and homeostasis.

In humans, Alu retrotransposon-derived RNAs are the most common sites of A-to-I editing.^7–9^ Alu genomic elements arose from a head-to-tail fusion of 7SL RNA, are ∼300 nucleotides in length, are unique to primates, and belong to the general class of short interspersed nuclear elements.^10–13^ About 1 million copies exist in the human genome, thus Alu retrotransposons make up about 10% of the total genome. Alu RNAs are transcribed by RNA polymerase 2 as part of a pre-mRNA and by RNA polymerase 3 as part of their normal life cycle. Because of their repetitive nature, Alu RNAs can form dsRNA structures that activate dsRNA sensors, such as TLR3, RIG-I and MDA5, resulting in innate immune activation including expression of interferons, interferon-stimulated genes (ISGs) and other cytokines and inflammatory mediators.^14–23^ A-to-I editing of Alu RNAs prevents their activation of dsRNA sensors and innate immune cell activation, thus representing another important function of A-to-I editing by ADAR enzymes. In fact, inactivating mutations in *ADAR* in humans result in one form of Aicardi-Goutières syndrome characterized by continuous innate immune activation producing severe encephalopathy that can lead to death or existence in a vegetative state.^20^

We have previously demonstrated loss of A-to-I editing in common inflammatory diseases including severe COVID-19 disease (both leukocytes and lung) and severe influenza (requiring hospitalization, leukocytes) as well as relapsing-remitting multiple sclerosis and inflammatory bowel diseases, ulcerative colitis and Crohn’s disease.^24–28^ These diseases are also associated with excessive innate immune cell activation believed to contribute to pathogenesis. In multiple sclerosis, Alu dsRNAs accumulate resulting in innate immune cell activation.^28^ Thus, loss of A-to-I editing, not as severe as is seen in Aicardi-Goutières syndrome, may also contribute to pathogenesis in these more common inflammatory diseases.

Alzheimer’s disease is also a common neurodegenerative encephalopathy resulting in disability and death and the most predominant form of dementia characterized by the accumulation of aggregated misfolded proteins including beta-amyloid plaques and neurofibrillary tangles.^29–31^ Of the different regions of the brain, both loss of total hippocampus (HPC) volume and breakdown of the blood–brain barrier in HPC are seen in humans with mild cognitive impairment and are some of the earliest changes associated with development of Alzheimer’s disease.^32–35^ In fact, the brain is one of the most vascularized human organs and total vascular cell density approximates that of total glial cell density.^36^ Recent studies also demonstrate presence of ongoing chronic inflammatory responses in brains of people with Alzheimer’s disease suggesting that innate immune activation may contribute to Alzheimer’s disease pathogenesis.^37,38^ As in studies described above, a source of innate immune activation may be loss of A-to-I editing and accumulation of Alu dsRNAs. Therefore, we sought to explore if A-to-I editing dysfunction existed in the human brain in Alzheimer’s disease and if so, determine if A-to-I editing dysfunction is present in multiple brain regions or localizes to specific brain regions. To explore this hypothesis, we analyzed whole-genome RNA sequencing (RNA-seq) FASTQ files to measure levels of A-to-I editing in both prefrontal cortex (CTX) and HPC obtained from patients with either normal cognition (NCI) or Alzheimer’s disease at the time of death. We find similar levels of A-to-I editing in NCI and Alzheimer’s disease CTX samples but a modest loss of A-to-I editing in Alzheimer’s disease HPC compared with NCI HPC. To explore these ideas further, we also analyzed CTX and HPC vasculature. Levels of A-to-I editing are similar between NCI and Alzheimer’s disease CTX vasculature but are markedly reduced in HPC vasculature. Taken together, our results demonstrate brain region-specific loss of A-to-I editing in Alzheimer’s disease, and this may contribute to observed innate immune activation in Alzheimer’s disease and associated pathogenesis.

## Materials and methods

### Study population and sample collection

We employed whole-genome RNA-seq FASTQ files obtained from the National Center for Biotechnology Information’s Gene Expression Omnibus, GEO. Briefly, these files originated from the following sample sets: (i) and (ii) analysis A-to-I editing in HPC and CTX vasculature RNA-seq files: post-mortem fresh-frozen HPC and superior frontal CTX tissues were obtained from the Stanford/VA/NIA Aging Clinical Research Center with institutional approvals and patient consent and HPC vasculature and CTX vasculature were isolated as described. Libraries were prepared using 10 ×  Genomics reagents and droplet-based single nuclei RNA-seq was performed; GEO accession number is GSE163577^36^; (iii) A-to-I editing in total CTX: samples of dorsolateral prefrontal CTX were obtained from the Mount Sinai Brain Bank, RNA isolated using Trizol, and libraries prepared using Illumina High-throughput TruSeq RNA sample preparation after depletion of ribosomal RNAs. Sequencing was performed using the Illumina HiSeq 2500 instrument, GEO accession number is GSE53699^39^; (iv) A-to-I editing in total HPC: brain tissues were obtained from the Chinese National Human Brain Bank for Development and Function and total HPC was dissected, RNA isolated, libraries prepared and sequencing performed using the Illumina HiSeq 2000; GEO accession number is GSE184942.^40^ Number of samples in each study population was as follows: (i) HPC vasculature; Alzheimer’s disease, #=7 (four females, three males), NCI, #=7 (four females, three males), (ii) CTX vasculature; Alzheimer’s disease, #=4 (two females, two males), NCI, #=4 (two females, two males), (iii) HPC; Alzheimer’s disease, #=5 (three females, two males), NCI, #=5, (three females, two males), (iv) CTX; Alzheimer’s disease, #=8 (four females, four males), NCI, #=8 (four females, four males). A clinical diagnosis of Alzheimer’s disease was employed to group patients into Alzheimer’s disease and NCI cohorts, for example, using the consortium to establish a registry for Alzheimer’s disease criteria.^41^

### A-to-I editing

We processed bulk RNA-seq FASTQ files including read trimming and employed the following workflow to identify endogenous RNA A-to-I–editing sites from paired FASTQ sequencing files essentially as previously described.^42^ The main identification tool was a python-based package called the SPRINT toolkit^43^ that accepts sequence files and produces text files with the following information for each edit site: (i) genomic location; (ii) type of edit (e.g. A-to-G; T-to-C), strand (+ or −); (iii) number of edits per site and total number of reads per site. Mathematica programmes were developed to synthesize data: numbers of samples in groups with shared editing sites, mean numbers of total reads and edits for each editing site, editing sites common and unique to group pairs, and editing sites per gene.^25^ This information was tied to an Alu database to annotate each site: gene locations [intronic, noncoding RNA, intergenic, 3′ and 5′ untranslated regions (UTRs)] and if editing sites were within Alu or non-Alu elements.^44^ To avoid sequencing errors, editing sites were only included in the analysis if total reads were greater than 5 and edit/read ratios were greater than 0.05. To create genome-wide A-to-I editing indices, we identified all A-to-I editing sites present in two or more samples in one study cohort and summed edit/read ratios for all editing sites across the genome within case or control cohorts. Gene expression levels from RNA-seq FASTQ files were determined using the DESeq2 R package as described.^45^ Both raw *P*-values and *P*-values after adjusting for multiple testing, false discovery rate (FDR), were determined.

### Synthesis and testing of Alu RNAs

Alu DNA sequences were from the GrCh37 (hg19) assembly. We designed unedited Alu DNA templates and changed A nucleotides edited in NCI HPC total vasculature but unedited in Alzheimer’s disease HPC total vasculature to G nucleotides as a mimic of A-to-I editing. Complete DNA sequences of unedited and edited MDM4 AluSg4 elements are shown in Supplementary Table 1. A SP6 promoter was added to the 5′ end and synthetic double-stranded DNA templates were obtained from IDT. RNA transcription was performed using Megascript SP6 (Invitrogen) essentially as previously described.^46^ THP-1 reporter cell lines (Invivogen) contain a stably integrated luciferase gene under the control of either an IFN-stimulated response element, ISRE or NF-κB response element. The human HMC3 microglial cell line was obtained from ATCC. THP-1 reporter or HMC3 cells were cultured in RPMI-1640 supplemented with 10% fetal bovine serum (HyClone), glutamine, and penn/strep at 37°C in a humidified atmosphere of 5% CO_2_ in air. Transfections were performed using Lipofectamine RNAiMAX (Thermo Fisher Scientific).^28^ Luciferase activity was determined after 24 h using luciferin substrate (Invivogen) and light emission measured with a TD20/20 luminometer. Gene expression measurements were performed essentially as previously described.^25,26^

### Statistical analysis

FDRs were determined to correct for multiple testing using the DESeq2 R package. Unpaired t tests with Welch’s correction were used to determine statistical significance for nonparametric data. The Kruskal–Wallis test or one-way ANOVA was used to compare two or more independent samples of equal or different sizes. Dunn’s multiple comparison test was used to identify means significant from others. The two-way ANOVA was used to compare mean differences between groups segregated by two independent variables. Statistical analysis was performed using GraphPad Prism.

### Data availability

The data that support the findings of this study are available from the senior author (T.M.A.) upon reasonable request.

## Results

### A-to-I editing in total CTX and total HPC

**Figure 1:**
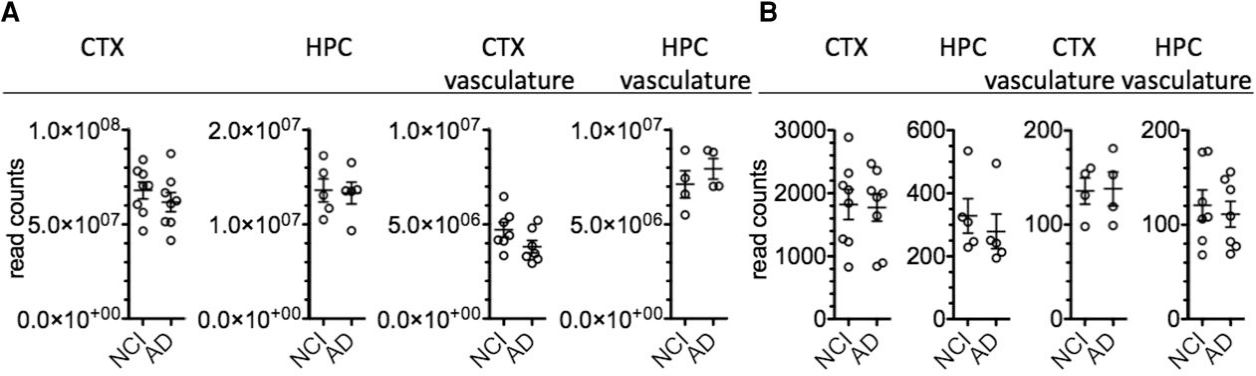
**Total read counts and HPRT1 read counts from RNA-seq FASTQ files of NCI and Alzheimer’s disease samples.** (**A**) Total read counts in total CTX, total HPC, CTX vasculature and HPC vasculature for NCI and Alzheimer’s disease cohorts. Each symbol represents an individual sample. (**B**) HPRT1 read counts in total CTX, total HPC, CTX vasculature and HPC vasculature for NCI and Alzheimer’s disease cohorts. Each symbol represents an individual sample. Differences in total read counts or HPRT read counts between NCI and Alzheimer’s disease samples in CTX, HPX, CTX vasculature, or HPC vasculature were not statistically significant, unpaired *t*-test with Welch’s correction.

With the goal of comparing A-to-I editing of endogenous RNAs among human brain tissues from people with Alzheimer’s disease and people with NCI at the time of death and brain donation, we obtained RNA-seq FASTQ files deposited in GEO derived from the following tissue sample sets: Alzheimer’s disease or NCI CTX, Alzheimer’s disease or NCI HPC, Alzheimer’s disease or NCI CTX vasculature, and Alzheimer’s disease or NCI HPC vasculature. As quality control steps, we determined total read counts present in each file. We found that all NCI and Alzheimer’s disease files within each cohort, CTX, HPC, CTX vasculature and HPC vasculature, had similar total read counts between NCI and Alzheimer’s disease cohorts (Fig. 1A). We also determined total read counts for the standard ‘housekeeping gene’, *HPRT1*, among NCI and Alzheimer’s disease files within the different cohorts. Again, we found that *HPRT1* read counts were similar among NCI and Alzheimer’s disease files within each cohort (Fig. 1B). Since, total RNA read counts and *HPRT1* read counts were similar between case-control cohorts, we concluded these RNA-seq FASTQ files were suitable to compare levels of RNA A-to-I editing among the different samples.

We employed the SPRINT software package integrated with the Alu database to determine levels of A-to-I editing of endogenous Alu RNAs.^43,44^ We first focused on total CTX and what we consider common editing sites. First, we identified A-to-I editing sites present in all NCI but not all Alzheimer’s disease samples or all Alzheimer’s disease samples but not all NCI samples and those editing sites shared between all NCI and Alzheimer’s disease samples and ranked them according to edit/read ratios. Using these criteria, we identified about 400 common editing sites present in all NCI samples but not all Alzheimer’s disease samples and about 500 common editing sites present in all Alzheimer’s disease samples but not all NCI samples (Supplementary Fig. 1A). We identified about 400 common editing sites shared between all NCI samples and all Alzheimer’s disease samples (Supplementary Fig. 1B). Average edit/read ratios at these common edited sites were not statistically different between NCI and Alzheimer’s disease cohorts (Supplementary Fig. 1C).

Next, we calculated a genome-wide editing index by identifying all edited sites with > 5 reads, edit/read ratios >0.05 and present in ≥2 samples in each cohort and multiplied average edit/read ratios by the number of samples with edits at each site to calculate an editing index per site. Using these criteria, we identified 15 000 editing sites in the NCI cohort and 12 000 editing sites in the Alzheimer’s disease cohort and the index/site ranged from 0.1 to >6 (Supplementary Fig. 1D). As above, average index/site was not statistically different between NCI and Alzheimer’s disease cohorts (Supplementary Fig. 1E). Taken together, we found similar numbers of editing sites and similar editing indices in total CTX between NCI and Alzheimer’s disease cohorts.

**Figure 2:**
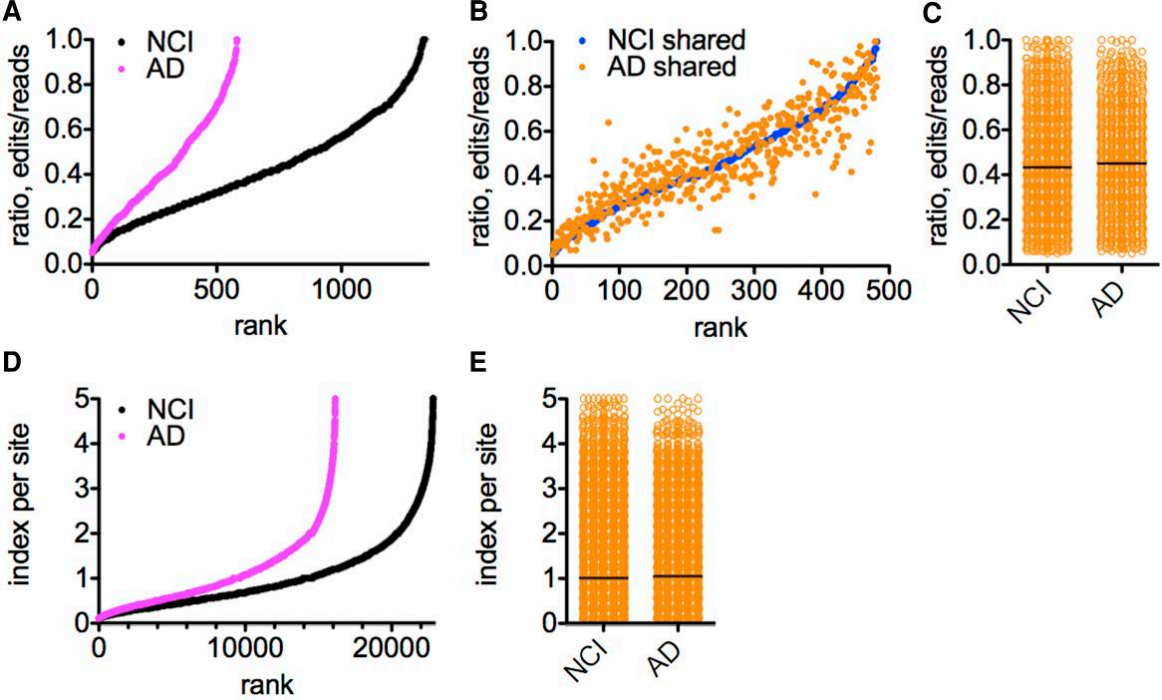
**Comparison of Alu RNA A-to-I editing in NCI and Alzheimer’s disease total HPC.** (**A**) Number of A-to-I editing sites present in either all HPC samples within the NCI cohort or all samples within the Alzheimer’s disease cohort. Y-axis is ratio of edits/reads at each edited site, X-axis is rank, *P* < 0.0001 c^2^ analysis comparing number of editing sites. (**B**) Number of editing sites shared between all HPC samples with the NCI and Alzheimer’s disease cohorts. Y-axis is the ratio of edits/reads at each edited site, X-axis is rank. (**C**) As in (**A**&**B**) except results are presented as a dot plot; line is the mean. *P* > 0.05, *t*-test with Welch’s correction comparing edit/read ratios. (**D**) NCI and Alzheimer’s disease genome-wide editing indices. Index at each site was determined by multiplying the edit/read ratio by the number of samples in each cohort with edits, X-axis; Y-axis is the rank, *P* < 0.0001 χ^2^ analysis comparing number of editing sites. (**E**) As in (**D**) except results are presented as a dot plot; line is the mean, *P* > 0.05, *t*-test with Welch’s correction comparing editing indices.

We employed a similar approach to compare A-to-I editing in NCI and Alzheimer’s disease total HPC. We found about 1400 common editing sites present in all NCI total HPC but only about 400 common editing sites present in all Alzheimer’s disease total HPC (Fig. 2A). We also identified about 500 common editing sites shared between all NCI and Alzheimer’s disease samples in total HPC (Fig. 2B). Average edit/read ratios at all common editing sites were not statistically different between NCI and Alzheimer’s disease cohorts (Fig. 2C).

As above, we calculated the genome-wide editing index and identified about 22 000 editing sites in NCI total HPC, but only about 15 000 total editing sites in Alzheimer’s disease total HPC using the above criteria (Fig. 2D). As above, average index/site was not statistically different between NCI and Alzheimer’s disease cohorts (Fig. 2E). Taken together, these results support the idea that there may be decreased A-to-I editing in Alzheimer’s disease HPC compared with NCI HPC.

### A-to-I editing in CTX vasculature and HPC vasculature

The brain is one of the most highly vascularized organs in the human body and brain vascular dysfunction contributes to a number of neurological disorders including neurodegenerative diseases such as Alzheimer’s disease.^32,36^ Therefore, we compared A-to-I editing in CTX vasculature between NCI and Alzheimer’s disease cohorts. We identified about 1400 common editing sites present in all NCI samples and a similar number of common editing sites present in all Alzheimer’s disease samples (Supplementary Fig. 2A). We also identified about 1200 common editing sites shared between all samples within both NCI and Alzheimer’s disease cohorts (Supplementary Fig. 2B). Average edit/read ratios at all common editing sites were not statistically different between NCI and Alzheimer’s disease cohorts (Supplementary Fig. 2C).

We also calculated the genome-wide editing index and identified 20 000 editing sites in samples from the NCI cohort and a similar number of editing sites in samples from the Alzheimer’s disease cohort (Supplementary Fig. 2D). As above, average index/site was not statistically different between NCI and Alzheimer’s disease cohorts (Supplementary Fig. 2E). Thus, we conclude that levels of A-to-I editing in CTX vasculature between NCI and Alzheimer’s disease cohorts are similar.

**Figure 3:**
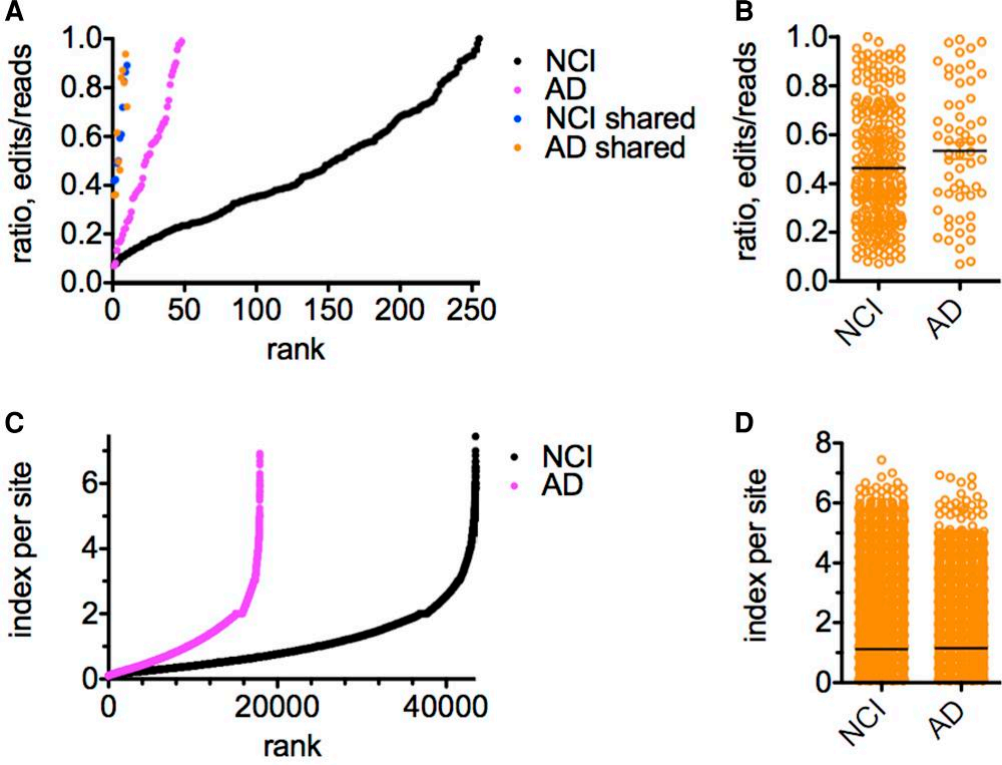
**Alzheimer’s disease-dependent changes in A-to-I editing in hippocampus vasculature.** (**A**) Number of A-to-I editing sites present in either all samples within the NCI cohort or all samples within the Alzheimer’s disease cohort or shared between all samples in both cohorts. Y-axis is ratio of edits/reads at each edited site, X-axis is rank. *P* < 0.0001, χ^2^ analysis comparing number of editing sites. (**B**) As in (**A**) except results are presented as a dot plot; line is the mean, *P* > 0.05. (**C**) NCI and Alzheimer’s disease genome-wide editing indices. Index at each site was determined by multiplying the edit/read ratio by the number of samples in each cohort with edits, X-axis; Y-axis is the rank. *P* < 0.0001, χ^2^ analysis (**D**) As in (**C**) except results are presented as a dot plot; line is the mean, *P* > 0.05.

We next compared A-to-I editing in HPC vasculature between NCI and Alzheimer’s disease cohorts employing a similar strategy as outlined above. We identified about 250 common editing sites present in all samples within the NCI cohort but not all samples within the Alzheimer’s disease cohort (Fig. 3A). We identified only about 50 common editing sites present in all samples in the Alzheimer’s disease cohort but not all samples within the NCI cohort. We identified only 10 common editing sites shared between all samples in both cohorts. Average edit/read ratios at all common editing sites were not statistically different between NCI and Alzheimer’s disease cohorts (Fig. 3B).

We also calculated the genome-wide editing index and identified about 40 000 editing sites in samples from the NCI cohort but only about 15 000 editing sites in the Alzheimer’s disease cohort (Fig. 3C). As above, average index/site ratios were not statistically different between NCI and Alzheimer’s disease cohorts (Fig. 3D). Thus, we conclude that levels of A-to-I editing in HPC vasculature were reduced in the Alzheimer’s disease cohort compared to the NCI cohort.

**Figure 4:**
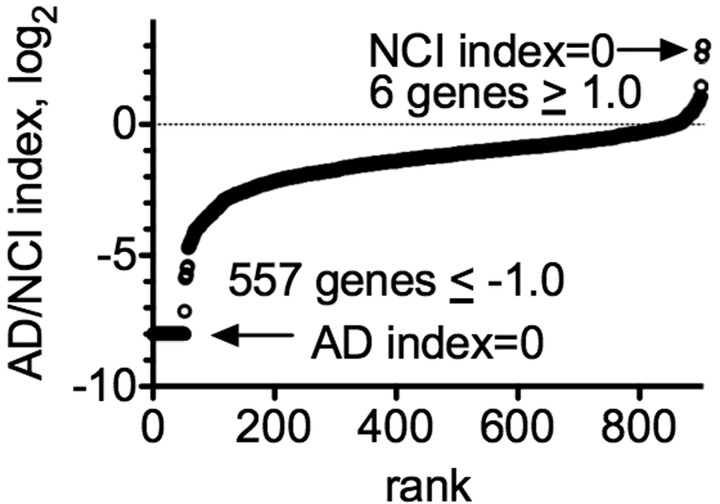
**Reduced A-to-I edits per gene in Alzheimer’s disease HPC vasculature.** Genes were identified with >5 edits per gene in >2 samples in either NCI or Alzheimer’s disease cohorts. Y-axis is Alzheimer’s disease/NCI index per gene, log2, X-axis is gene rank from lowest to highest AD/NCI index. NCI or Alzheimer’s disease index = 0 indicates 0 edits per gene detected in either NCI or Alzheimer’s disease cohorts, respectively.

The majority, >90%, of all A-to-I editing sites we identified were found in genic regions, either introns or 5′ and 3′ UTRs and were within Alu elements. Therefore, as another measure of loss of editing in Alzheimer’s disease HPC vasculature, we determined the editing index per gene in NCI and Alzheimer’s disease cohorts and expressed these data as Alzheimer’s disease/NCI ratios, log_2_. To do so, we used the same criteria employed above, total reads >5, proportion of edits/reads >0.05 to define an editing site, and nucleotide sites were edited in ≥2 samples in either cohort. For this analysis, we identified all genes with >5 editing sites per gene in either NCI or Alzheimer’s disease. We found that the majority of genes with >5 editing sites per gene displayed reduced editing in Alzheimer’s disease compared with NCI (Fig. 4). We only identified a small number of genes with increased editing in Alzheimer’s disease compared with NCI. Thus, using several different criteria, we found reduced A-to-I editing of RNAs containing Alu elements in Alzheimer’s disease HPC vasculature compared with NCI HPC vasculature.

We considered that a possible explanation for the observed deficiencies in editing in Alzheimer’s disease HPC vasculature compared with NCI HPC vasculature might be differences in cell composition of the HPC vasculature arising from cell death or other means. As part of the previous single-cell RNA-seq studies,^36^ genes were identified that were preferentially expressed in one lineage, calculated be determining the percent of cells within a given cluster where the expressed gene was detected, termed pct1, compared with the frequency of expression in all the other lineages, termed pct2. We identified genes with pct1/pct2 ratios >10 for each cell lineage (Supplementary Fig. 3A, D). We determined expression levels of each of these genes from the bulk RNA-seq data, expressed as NCI/ Alzheimer’s disease ratios, log_2_. We also determined adjusted *P*-values for each of these lineage-defining genes comparing NCI and Alzheimer’s disease. We did not find uniform substantial increases or decreases in expression of these potential lineage-defining genes in any of the populations analyzed in the NCI or Alzheimer’s disease cohorts (Supplementary Fig. 3B). We did find that astrocyte, ependymal and oligodendrocyte lineage-defining genes tended to show decreased expression in the NCI cohort compared with the Alzheimer’s disease cohort and pericyte lineage-defining genes tended to show increased expression in the NCI cohort compared to the Alzheimer’s disease cohort. However, these differences were small and differences were not statistically significant between NCI and Alzheimer’s disease cohorts (Supplementary Fig. 3C). From these analyses, we interpret that substantial changes in cellular composition in HPC vasculature do not explain the observed differences in A-to-I editing between NCI and Alzheimer’s disease cohorts.

**Figure 5:**
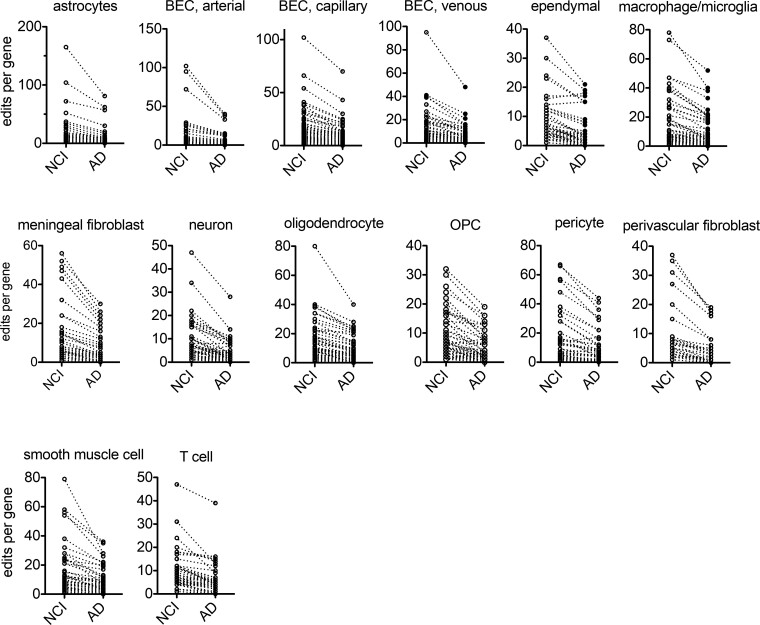
**Genes that exhibit cell lineage-specific expression in HPC vasculature show preferential loss of A-to-I editing in Alzheimer’s disease.** Lineage-specific genes in each cell cluster were identified by the pct1/pct2 ratio; pct1 is the percentage of cells in a cluster where the expressed gene is detected, pct2 is the percentage of cells in all other clusters where the gene is detected; upper three rows (see Yang et al. for details, see also supplementary file 1). Y-axis is the A-to-I editing index (see Methods). X-axis shows results for the NCI and Alzheimer’s disease groups.

We thought we could use a similar strategy to determine if defects in A-to-I editing in HPC vasculature may be localized to one or more cell lineages that make up the brain vasculature or if loss of A-to-I editing was observed in most or all of these cell lineages. As described above, genes were previously classified as lineage-specific genes based upon their pct1/pct2 ratios (Supplementary File 1).^36^ Therefore, we asked if genes that defined specific HPC vasculature cell lineages were differentially edited in Alzheimer’s disease and NCI groups. To do so, we calculated the ratio of edits per gene for each lineage-defining gene in Alzheimer’s disease and NCI HPC vasculature groups. Our rationale was that if Alzheimer’s disease-dependent loss of A-to-I editing was restricted to a single or small number of cell lineages in the vasculature, then the genes that define a given lineage should exhibit greater loss of A-to-I editing in Alzheimer’s disease than NCI while genes that define other lineages should exhibit equivalent levels of A-to-I editing between Alzheimer’s disease and NCI groups if loss of editing was limited to a specific cell lineage. The alternative was that we would observe a general loss of A-to-I editing in the majority of genes that defined all unique cell lineages. To perform this comparison, we calculated number of edits found in genes that defined the different cell lineages that make up the HPC vasculature in both the Alzheimer’s disease and NCI groups and plotted this ratio versus the gene rank from lowest to highest Alzheimer’s disease/NCI editing ratio per gene. Using this approach, we found that all cell lineages of the HPC vasculature demonstrated a quantitatively similar overall loss of editing at lineage-defining genes (Fig. 5, summarized in Table 1). Thus, it appears that there is an overall loss of A-to-I editing in all cell lineages of the HPC vasculature rather than a selective loss of A-to-I editing in a small number of cell lineages making up the HPC vasculature in Alzheimer’s disease.

**Table 1:** Summary of results presented in Fig. 5

	#Samples		#Genes	Mean ± SEM		Unpaired Welch’s *t*-test		
Cell type	NCI	Alzheimer’s disease		NCI	Alzheimer’s disease	*P*	t	Df
Astrocyte	7	7	83	12.3 ± 2.5	6.2 ± 1.4	0.03	2.1	129
BEC, arterial	7	7	34	16.8 ± 4.2	7.6 ± 1.8	0.04	2.0	44
BEC, capillary	7	7	74	13.7 ± 1.9	8.5 ± 1.3	0.02	2.3	125
BEC, venous	7	7	46	13.9 ± 2.4	7.3 ± 1.2	0.01	2.5	67
Ependymal	7	7	58	6.4 ± 1.0	3.7 ± 0.7	0.02	2.2	104
Microglia/macrophage	7	7	78	12.1 ± 1.7	6.7 ± 1.2	0.01	2.5	132
Meningeal fibroblast	7	7	47	11.7 ± 2.2	5.7 ± 1.2	0.02	2.4	69
Neuron	7	7	84	5.4 ± 0.8	2.6 ± 0.5	0.003	3.0	128
Oligodendrocyte	7	7	86	10.7 ± 1.3	6.4 ± 0.9	0.008	2.7	144
OPC	7	7	75	6.4 ± 0.9	3.2 ± 0.5	0.002	3.1	121
pericyte	7	7	54	13.5 ± 2.3	7.7 ± 1.5	0.03	2.1	89
Perivascular fibroblast	7	7	34	8.3 ± 1.7	3.9 ± 0.9	0.03	2.2	51
Smooth muscle cell	7	7	43	16.6 ± 2.6	9.2 ± 1.4	0.02	2.5	64
T cell	7	7	50	8.5 ± 1.2	5.4 ± 0.9	0.04	2.1	92

Cell type, number of samples in NCI and Alzheimer’s disease groups, number of genes used to define each cell lineage, mean ± SEM of combined A-to-I editing index for all genes used to define each cell type, and statistical analysis using unpaired Welch’s *t*-test: *P*-value, *t* statistic, df (degrees of freedom).

### Differential expression of genes that encode RNA-binding proteins in Alzheimer’s disease

ADAR1 and ADAR2, encoded by *ADAR* and *ADARB1* respectively, are the major A-to-I editing enzymes in eukaryotic cells.^7^ Recent studies demonstrated that a number of additional RNA-binding proteins (RBPs) also contribute to overall levels of genome-wide A-to-I editing.^47^ To perform these studies, genes that encode ADAR1 and ADAR2, as well as additional RBPs, were individually subjected to siRNA knockdown in both K562 and HepG2 cells and levels of genome-wide A-to-I editing determined. For example, loss of *ADAR1* via siRNA knockdown in both K562 and HepG2 cells resulted in >50% loss of genome-wide A-to-I editing. Knockdown of *XRCC6* or *FXR1* also resulted in a similar loss of A-to-I editing in these same cell lines.^47^

**Figure 6:**
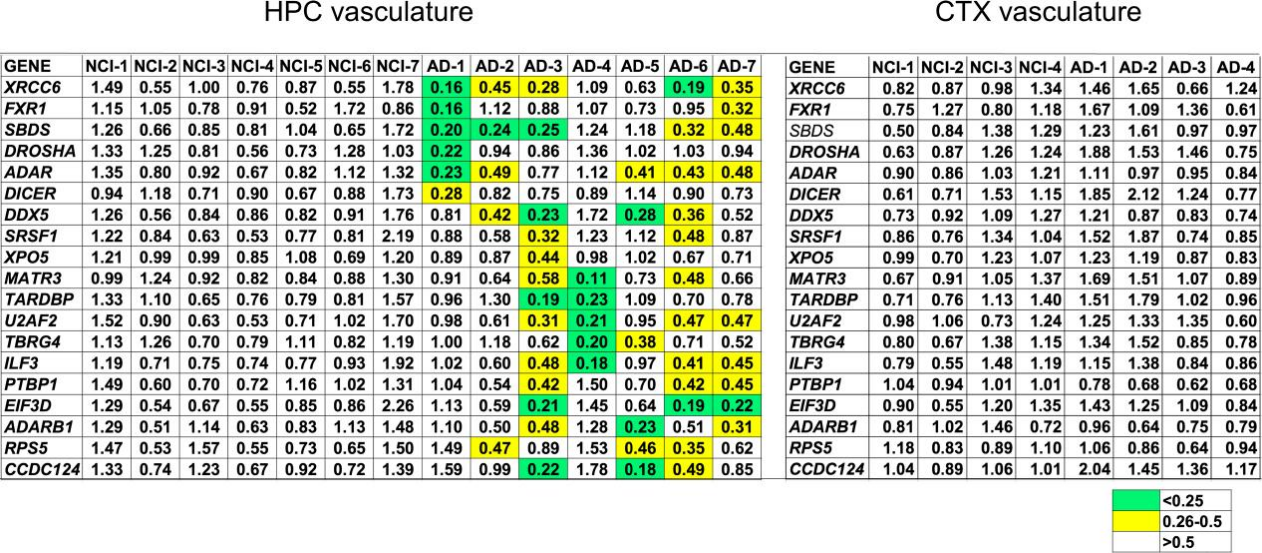
**Expression levels of genes encoding RBPs known to affect A-to-I editing in NCI and Alzheimer’s disease cohorts.** From RNA-seq FASTQ files, read counts were determined for each of the indicated expressed genes. An Alzheimer’s disease/NCI ratio for each gene for each sample was calculated by dividing the read count for each sample by the NCI average read count to determine Alzheimer’s disease and NCI expression ratios. Individual expression ratios are shown; key to colour coding is shown. Results from HPC vasculature and CTX vasculature are shown.

We compared expression levels of *ADAR*, *ADARB1*, and these other RBP encoding genes in NCI and Alzheimer’s disease cohorts in both HPC vasculature where we observe substantial loss of A-to-I editing in the Alzheimer’s disease cohort and CTX vasculature where we do not observe substantial loss of A-to-I editing in the Alzheimer’s disease cohort. First, we determined average Alzheimer’s disease/NCI expression ratios and adjusted *P*-values in both HPC vasculature and CTX vasculature for each of these genes using the DESeq2 package. We did not observe significant differences in expression levels of any of these genes comparing either all NCI and Alzheimer’s disease HPC vasculature samples or all NCI and Alzheimer’s disease CTX vasculature samples (adjusted *P* > 0.05, not shown). As an alternative approach, we examined expression of genes encoding these RBPs in each sample of the cohorts under study. To do so, we used the expression level of each of the test genes in each sample as the numerator and the average expression ratio of the test gene in the relevant NCI cohort as the denominator. Using this approach, we identified numerous genes encoding RBPs required for efficient A-to-I editing that displayed reduced expression in one or more Alzheimer’s disease sample in the HPC vasculature group but there was not uniform loss of a single RBP in the Alzheimer’s disease sample set (Fig. 6). For example, we found that levels of *ADAR*, *XRCC6* and *SBDS* were reduced in 5 of 7 Alzheimer’s disease samples and levels of *DDX5*, *U2AF2*, *ILF3* were reduced in 4 of 7 Alzheimer’s disease NPC vasculature samples by greater than 50%. Using a cut-off Alzheimer’s disease/NCI ratio of 0.5, we did not find a single NCI sample in the HPC vasculature group in which these genes were under-expressed. We performed a similar comparison between NCI and Alzheimer’s disease samples in the CTX vasculature group and did not identify a single RBP with an Alzheimer’s disease/NCI ratio <0.5 or >2.0. Thus, we found that different RBPs required for efficient A-to-I editing displayed reduced expression in Alzheimer’s disease HPC vasculature samples where we also observe reduced A-to-I editing. Genes encoding these same RBPs were not under-expressed in other cohorts where we do not observe reduced levels of A-to-I editing.

### A-to-I editing of Alu RNAs lowers their ability to stimulate IRF and NF-κB signalling

**Figure 7:**
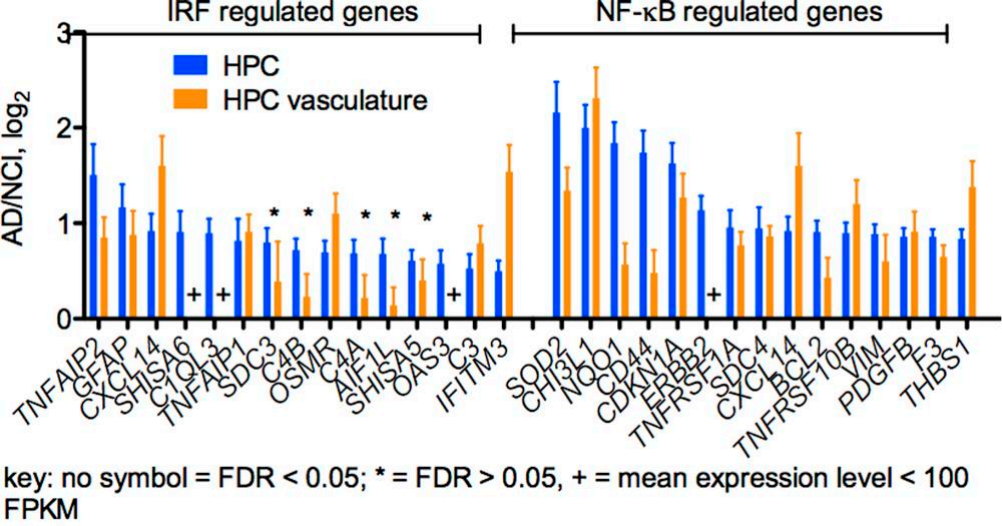
**Expression levels of ISGs and NF-κB regulated genes are elevated in the HPC Alzheimer’s disease and HPC vasculature groups compared with the HPC NCI group and the HPC vasculature NCI group, respectively.** Y-axis is the average FPKM Alzheimer’s disease/NCI ratio, log_2_ for indicated genes determined using DESeq2, HPC (Alzheimer’s disease #=5, NCI #=5), HPC vasculature groups (Alzheimer’s disease #=7, NCI #=7). Genes are separated into IRF regulated genes (ISGs, left) or NF-κB regulated genes. *P* < 0.0001, two-way ANOVA for each category. Error bars are the log fold change standard error determined using DESeq2. Benjamini Hochberg adjusted *P*-values (FDR) were also determined using DESeq2 and grouped as follows: FDR < 0.05 (no symbol), FDR > 0.05 (*).

We also compared expression levels of known ISGs^48^ and NF-κB regulated genes^49^ in Alzheimer’s disease and NCI HPC and HPC vasculature. We found that both ISGs and NF-κB regulated genes were over-expressed in Alzheimer’s disease total HPC and Alzheimer’s disease HPC vasculature compared to NCI HPC and NCI HPC vasculature, respectively, thus providing a correlate to the decreased levels of A-to-I editing observed in Alzheimer’s disease (Fig. 7).

**Figure 8:**
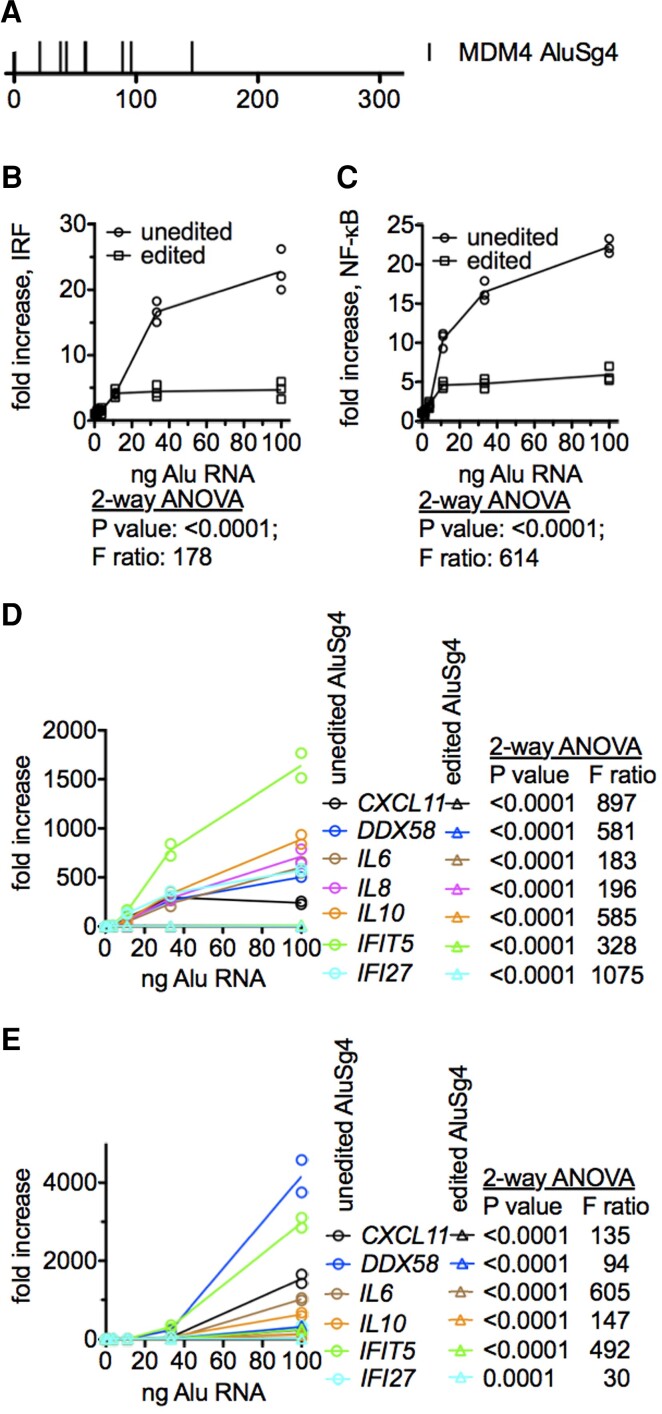
**A-to-I editing of Alu RNAs lowers their ability to stimulate IRF and NF-κB signalling.** (**A**) Locations of A-to-I edits present in the HPC vasculature NCI cohort but absent in HPC vasculature Alzheimer’s disease cohort in the AluSg4 element in *MDM4*. (**B**&**C**) Activation of IRF (**B**) and NF-κB (**C**) transcriptional responses by unedited (open circles) and edited (open squares) *MDM4* AluSg4 RNA; mean ± SD, Y-axis is fold increase in luciferase expression relative to mock-transfected controls. Cultures were performed in triplicate, (open circles and open squares. Lines show averages. B&C: *P* < 0.0001, two-way ANOVA comparing stimulations by unedited and edited AluSg4. F ratios are also shown. (**D**) THP-1 cells (duplicate cultures) were transfected with *MDM4* AluSg4 RNA as in (**B**). After 24 h of culture, levels of indicated genes were determined by RT-PCR, expression differences of each measured gene induced by unedited and edited *MDM4* AluSg4 were significant Statistical analysis was performed using 2-way ANOVA. *P*-values and F ratios for differences in induction of each gene are shown. (**E**) As in (D), except experiments were performed using the human HMC3 microglial cell line (duplicate cultures). Statistical analysis was performed using 2-way ANOVA. *P*-values and F ratios for differences in induction of each gene are shown.

The vast majority (>90%) of A-to-I editing sites we identified were located in Alu RNAs. Unedited Alu RNAs can form dsRNAs recognized by dsRNA sensors, RIG-I, MDA5, TLR3 and others and stimulate IRF and NF-κB transcriptional responses.^23,25,26^ We identified numerous highly expressed Alu RNAs that were edited in the NCI HPC vasculature cohort but were unedited in the Alzheimer’s disease HPC vasculature cohort. One example was an AluSg4 element located in the *MDM4* gene that encodes a p53 binding protein that inhibits p53 function. We transcribed the AluSG4 RNAs from double-stranded DNA templates. As a model of edited Alu RNAs, we replaced edited A’s found in the NCI AluSg4 RNA with G’s (see Supplementary Table 1 for DNA sequences of unedited and edited templates). In general, we found that only 6–8 A’s were edited to I’s in the NCI Alu RNAs and these A’s were unedited in the Alzheimer’s disease cohort (the AluSg4 example is shown, Fig. 8A). We tested ability of these unedited and edited Alu RNAs to stimulate IRF and NF-κB transcriptional pathways using THP-1 reporter cells. We found that the unedited Alu RNA was a potent activator of both IRF and NF-κB transcriptional pathways (Fig. 8 B, C). In contrast, the edited Alu RNA displayed only weak agonist activity. We also asked if unedited and edited Alu RNAs induced expression of known ISGs and known NF-κB induced genes in THP-1 cells that are known to be induced in THP-1 cells by type 1 IFNs.^25,26^ The unedited Alu RNA was a potent activator of both known ISGs, *DDX58*, *IFIT5*, *IFI27*, and known NF-κB regulated genes, *CXCL11*, *IL6*, *IL8* and *IL10*, while the edited Alu RNA only weakly induced these genes (Fig. 8D). We also tested induction of these genes in a second human myeloid cell line, the microglial cell line, HMC3, and obtained similar results (Fig. 8E). Thus, unedited Alu RNAs found in the Alzheimer’s disease HPC vasculature cohort were potent activators of both IRF and NF-κB pathways while edited Alu RNAs, as found in the NCI cohort, were only weak agonists of these pathways.

## Discussion

Our studies demonstrate a substantial loss of A-to-I editing in HPC vasculature in Alzheimer’s disease and a modest loss of editing in total HPC in Alzheimer’s disease, which may also reflect A-to-I editing differences in HPC vasculature. In contrast, levels of A-to-I editing in CTX, either total or vasculature are not substantially different in NCI or Alzheimer’s disease. Our analysis of expression of genes that define the different cell lineages present in the vasculature suggest that loss of A-to-I editing in HPC vasculature in response to Alzheimer’s disease results from relatively uniform loss of A-to-I editing in all cell lineages that make up the HPC vasculature rather than presence of affected and unaffected cell lineages. Levels of A-to-I editing are not only affected by levels and activity of the ADAR family of enzymes but are also affected by numerous other RBPs. We do not find uniform loss or gain of a single gene encoding one RBP that regulates A-to-I editing in Alzheimer’s disease HPC vasculature. Rather, we see differential expression of multiple genes that encode these RBPs across the Alzheimer’s disease population. Reduced A-to-I editing in HPC and HPC vasculature is also associated with increased expression of ISGs and NF-κB regulated genes. In a model culture system, examples of unedited Alu RNAs as found in Alzheimer’s disease HPC vasculature are potent innate immune activators while edited Alu RNAs as found in NCI HPC vasculature only weakly activate innate immune responses. Thus, observed loss of A-to-I editing in Alzheimer’s disease may contribute to innate immune activation and possibly other associated Alzheimer’s disease pathologies.

Among the brain regions we sampled, the most pronounced loss of A-to-I editing is found in Alzheimer’s disease HPC vasculature. In an independent sample set, modest loss of A-to-I editing is observed in Alzheimer’s disease total HPC. At this point, we do not know if loss of A-to-I editing is restricted to Alzheimer’s disease HPC vasculature or may extend to other regions of the HPC. The HPC is one of the earliest affected brain regions in Alzheimer’s disease and loss of HPC volume is a good predictor of presence of Alzheimer’s disease.^35^ HPC dysfunction is also thought to contribute to memory impairment, a core feature of Alzheimer’s disease.^34^ Further, hallmarks of Alzheimer’s disease including neurofibrillary tangles, hyperphosphorylated tau proteins, amyloid plaques, and loss of neurons accumulate in the HPC. Breakdown of the HPC vasculature and blood–brain barrier is also one of the first brain dysfunctions found in people with age-associated mild cognitive impairment as well as Alzheimer’s disease.^32,33,50,51^

HPC vasculature is composed of multiple cell lineages including astrocytes, endothelial cells, ependymal, fibroblasts, neurons, oligodendrocytes, pericytes, smooth muscle cells and T cells.^36^ Each of these cell lineages selectively express certain genes that are only weakly expressed by the other cell lineages that make up the HPC vasculature. It seemed to us that this could be employed as a means to infer whether loss of A-to-I editing in Alzheimer’s disease HPC vasculature is cell type-specific or is a general property of the majority of cell lineages that make up HPC vasculature. Patterns of Alzheimer’s disease-dependent loss or gain of editing of these lineage-defining genes are relatively similar for each cell lineage and relatively similar to the overall loss of A-to-I editing found in Alzheimer’s disease HPC vasculature. Our conclusion is that loss of A-to-I editing in HPC vasculature as a result of Alzheimer’s disease represents an overall loss of editing in the majority of cell types present within the vasculature as opposed to a localized loss of editing in a small number of cell lineages that make up HPC vasculature.

On the other hand, A-to-I editing in CTX vasculature does not appear to be altered by presence of Alzheimer’s disease and analytic procedures for cell isolation, preparation of libraries, RNA-seq procedures and analysis of A-to-I editing of NCI and Alzheimer’s disease HPC and CTX vasculature samples were identical.^36^ Further, levels of A-to-I editing in NCI and Alzheimer’s disease total CTX do not appear to be markedly different. Additional studies will be required to determine if other brain regions also have altered A-to-I editing in Alzheimer’s disease compared to NCI. Additional studies will also be required to determine if changes in A-to-I editing are found in other neurodegenerative diseases.

ADAR1 and ADAR2 are the known human enzymes that catalyze A-to-I editing.^6,17,20^ All ADARs share a common dsRNA binding domain and a catalytic deaminase domain. In addition to ADARs, a number of other RBPs are known to either positively or negatively impact editing. Mechanisms of action of different RBPs include sequestration of dsRNAs to prevent editing. Ro60 and TDP-43 are two examples.^52,53^ Loss of Ro60 expression or function has also been implicated in changes in A-to-I editing in multiple sclerosis.^54^ In addition, RBPs, such as DROSHA directly interact with ADAR to affect A-to-I editing.^47^ By examining our RNA-seq expression data for levels of different RBPs, we do not find uniform loss or gain of mRNAs that encode *ADAR1*, *ADAR2* or other RBPs that either positively or negatively impact A-to-I editing in Alzheimer’s disease HPC or CTX vasculature compared to NCI HPC or CTX vasculature. Rather, each Alzheimer’s disease HPC vasculature sample we analyzed displays reduced expression of two or more RBPs, including *ADAR1*, that positively affect A-to-I editing. This is not the case for CTX vasculature. Thus, one interpretation of these results is that loss of different RBPs in different Alzheimer’s disease HPC vasculature samples may cause the observed loss of A-to-I editing in Alzheimer’s disease HPC vasculature. Whether these changes may result from genetic or environmental factors is not known. Over 40 distinct Alzheimer’s disease genetic risk factors have been identified through extensive genome-wide association studies.^55,56^ None of these genetic loci are associated with genes that encode RBPs known to influence A-to-I editing. However, many of the RBPs under-expressed in Alzheimer’s disease HPC vasculature do play important roles in neurological health and neurodegenerative diseases including *ADAR*, *XRCC6*, *U2AF2* and *ILF3*.^57–59^ For example, the protein products of *DDX5*, *U2AF2* and *ILF3* play important roles in Tau protein splicing and biology.^59–61^ A major environmental factor that results in loss of A-to-I editing is viral infection, including both acute and chronic infection in diverse tissues and by infection with either single stranded or dsRNA viruses.^25,26,62^ Whether this may imply presence or absence of viral infection or differences in the host response to infection is unknown.

The brain is generally considered to have the highest levels of A-to-I editing among human tissues or organs suggesting that A-to-I editing is critical for normal brain development and homeostasis.^4,5^ In general terms, two major functions of A-to-I editing include either loss or gain of microRNA binding sites in mRNAs that can affect mRNA stability and translation and alterations in pre-mRNA splicing; both of which might be important for transcriptome diversification.^63^ A third major function is A-to-I editing of Alu dsRNAs.^17^ Alu dsRNAs, if unedited, result in innate immune activation via stimulation of dsRNA sensors and pervasive activation of IRF and NF-κB transcriptional responses producing something akin to an ‘anti-viral response’.^14,23^ One example is the *MDM4* AluSg4 RNA that is edited in NCI HPC vasculature and unedited in Alzheimer’s disease HPC vasculature. The unedited form is a potent activator of both IRF and NF-κB transcriptional responses and induces genes known to be regulated by these two transcription factors while the edited form only weakly activates these responses. In fact, recent studies investigating both human brain tissues and mouse models suggest that type 1 interferon responses drive neuro-inflammation and synapse loss in Alzheimer’s disease, thus perhaps supporting a causal role for viral or bacterial infection in development of Alzheimer’s disease.^37,38^ Our results support this Pathogen Hypothesis but argue that the problem is internal; disrupted A-to-I editing leads to accumulation of potentially pathogenic quantities of unedited Alu dsRNAs triggering dsRNA sensors resulting in strong innate immune activation.

Alzheimer’s disease is a neurodegenerative disorder affecting the elderly while multiple sclerosis is a neurodegenerative disease typically with onset as a young adult. Both Alzheimer’s disease and multiple sclerosis are associated with dysregulation of the immune system including both the innate and adaptive arms.^64^ Dysregulation of RNA processing or dynamics is also observed in Alzheimer’s disease and may contribute to Alzheimer’s disease pathogenesis.^65^ Similar to results presented here, we have also found loss of A-to-I editing in multiple sclerosis and accumulation of Alu dsRNAs recognized by endogenous dsRNA sensors resulting in strong activation of the innate arm of the immune system.^27,28^ We have also found substantial dysfunction of RNA processing or dynamics in multiple sclerosis and this may also arise from defective A-to-I editing.^54^ As such, it is tempting to speculate that loss of A-to-I editing may contribute to neurodegeneration that is common to both Alzheimer’s disease and multiple sclerosis.

Although rather speculative, our results raise the possibility that RNA editing based therapeutics may be useful for the treatment of Alzheimer’s disease and if existing therapeutics might be re-purposed to restore RNA editing in Alzheimer’s disease. For example, the ADAR protein undergoes ubiquitination and degradation by the proteasome in response to viral infection.^66^ It is reasonable to speculate that proteasome inhibition may increase ADAR levels and restore A-to-I editing in Alzheimer’s disease HPC vasculature. Currently, proteasome inhibitors are in clinical use including bortezomib (BTZ, Velcade^®^), carfilzomib (CFZ, Kyprolis), and ixazomib (IXZ, Ninlaro^®^), mostly in the field of oncology.^67^ These proteasome inhibitors may be re-purposed to prevent or reverse cognitive decline in Alzheimer’s disease. Second, both ADAR1p110 and ADAR2 are phosphorylated by AKT kinase and this phosphorylation reduces A-to-I editing catalyzed by ADAR in the range of 50–100%.^68,69^ Selective AKT inhibitors have also been developed, including capivasertib, ipatasertib, and are in Phase I and Phase II clinical trials in fields of oncology.^70^ It is also tempting to speculate that inhibition of ADAR phosphorylation by AKT inhibitors may restore Alzheimer’s disease -dependent loss of A-to-I editing in HPC vasculature and if this may potentially slow or reverse cognitive decline in Alzheimer’s disease. A better understanding of mechanisms that underlie Alzheimer’s disease -associated loss of A-to-I editing in HPC vasculature and functional consequences of loss of editing may point to additional therapeutic strategies that might be pursued to slow or reverse cognitive decline resulting from Alzheimer’s disease.

## Supplementary Material

fcac238_Supplementary_Data

## Abbreviations

ADAR=: adenosine deaminase specific for double-stranded RNA
A-to-I editing=: adenosine deamination to inosine in double-stranded RNA
CTX=: prefrontal cortex
dsRNA=: double-stranded RNA
HPC=: hippocampus
NCI=: tissue samples from people with normal cognition
RBPs=: RNA-binding proteins
