# Supplementary material for: Reduced RNA adenosine-to-inosine editing in hippocampus vasculature associated with Alzheimer’s disease
Source: Brain Commun. 2022 Sep 22;4(5):fcac238. doi: 10.1093/braincomms/fcac238 (PMC9527665; doi:10.1093/braincomms/fcac238)
Supplement: fcac238_Supplementary_Data [file fcac238_supplementary_data.zip › SupFile1.pdf]

Supplementary File 1. Genes selectively expressed in the indicated cell lineages in HPC vasculature, page 1, from Yang et. al.

Cell Type: Astrocyte

Gene	pct.1	pct.2	Gene	pct.1	pct.2
AGBL4	0.315	0.043	SHROOM3	0.278	0.015
AHCYL1	0.453	0.083	CLU	0.466	0.088
ARHGEF4	0.492	0.027	HPSE2	0.278	0.02
CDHR3	0.309	0.019	NTM	0.826	0.285
CNTN1	0.674	0.107	STK33	0.291	0.034
CRB1	0.356	0.05	DNAH7	0.246	0.019
DAAM1	0.402	0.08	LRP1B	0.861	0.235
DAPK1	0.294	0.018	PDE4DIP	0.625	0.113
FAT3	0.301	0.021	SLC1A3	0.675	0.27
GABRB1	0.495	0.037	TRPM3	0.631	0.046
MGST1	0.299	0.011	CTNND2	0.904	0.204
PTPRZ1	0.465	0.045	DDAH1	0.296	0.035
SLC38A1	0.198	0.028	RGS20	0.257	0.006
ST8SIA1	0.269	0.034	SLC7A11	0.438	0.13
ADCY2	0.774	0.054	TPST1	0.296	0.077
DCLK1	0.458	0.066	ITPKB	0.549	0.141
LRIG1	0.451	0.102	NEBL	0.821	0.362
RPS6KA2	0.648	0.281	PALLD	0.377	0.042
SLC1A2	0.678	0.023	RANBP3L	0.35	0.047
SLC4A4	0.428	0.042	DOCK7	0.479	0.108
TNIK	0.656	0.117	NPL	0.299	0.022
APC	0.444	0.105	CAMK2G	0.317	0.048
CABLES1	0.22	0.03	GNA14	0.242	0.034
DCLK2	0.569	0.101	PRKCA	0.74	0.265
DTNA	0.892	0.176	RFX2	0.283	0.036
ITGB4	0.204	0.015	TRPS1	0.712	0.264
NHSL1	0.429	0.11	TNRC6A	0.705	0.287
NTRK2	0.727	0.121	CTNNA2	0.889	0.16
RYR3	0.642	0.043	NPAS3	0.951	0.349
SLC25A18	0.377	0.03	OPHN1	0.485	0.125
WWC1	0.258	0.012	DGKG	0.344	0.055
ADCY8	0.309	0.016	GPM6A	0.853	0.12
ASPH	0.45	0.101	NRG3	0.964	0.152
CD44	0.376	0.035	SAMD4A	0.454	0.138
COL21A1	0.219	0.02	TPD52L1	0.477	0.047
KCNMA1	0.557	0.101	ERBB4	0.795	0.281
MRVI1	0.217	0.019	MMD2	0.203	0.014
ZHX3	0.473	0.128	ACOT11	0.267	0.021
ZNRF3	0.515	0.097	LSAMP	0.968	0.338
ATP13A4	0.359	0.028	ABLIM1	0.638	0.191
EYA2	0.287	0.015	DPP10	0.88	0.159
FMN2	0.747	0.053	PPP2R2B	0.891	0.335
GLIS3	0.794	0.195	MAP3K5	0.455	0.123
KCNN3	0.333	0.016	AHCYL2	0.473	0.168
MAPK10	0.704	0.247	HIF3A	0.572	0.187
MAPK4	0.365	0.021	SORBS1	0.86	0.317
NRXN1	0.57	0.105	PITPNC1	0.837	0.342
SFXN5	0.433	0.053			

Cell Type: BEC, Arterial

Gene	pct.1	pct.2
CCDC85A	0.418	0.138
COL8A1	0.157	0.027
EMCN	0.295	0.077
PDZRN3	0.571	0.267
PALMD	0.275	0.075
ADAMTS6	0.19	0.049
ITGA1	0.469	0.183
ANO2	0.561	0.182
BMP6	0.33	0.058
ERG	0.487	0.139
KYNU	0.144	0.012
RNF144B	0.459	0.111
ST6GALNAC3	0.951	0.457
THSD7A	0.591	0.16
CPNE8	0.507	0.188
CRIM1	0.572	0.253
MCTP1	0.407	0.092
PELI1	0.336	0.112
EXOC6	0.416	0.152
MECOM	0.847	0.226
CHSY1	0.448	0.158
GFOD1	0.359	0.084
PECAM1	0.38	0.094
PLXNA2	0.389	0.122
PTPRB	0.411	0.117
PTPRM	0.793	0.374
ELOVL7	0.797	0.256
CMTM8	0.375	0.1
TMTC1	0.546	0.218
THSD4	0.672	0.181
FLNB	0.35	0.095
FLT1	0.87	0.294
SEC14L1	0.507	0.198
LDB2	0.556	0.173
PLCG2	0.225	0.026
ARL15	0.916	0.289
BMPR2	0.587	0.27
PTPRG	0.929	0.551

Cell Type: BEC, Capillary

Gene	pct.1	pct.2	Gene	pct.1	pct.2
ANXA3	0.221	0.029	SHANK3	0.245	0.051
ATP7B	0.198	0.027	ADIPOR2	0.65	0.253
BSG	0.628	0.203	CHSY1	0.423	0.13
CCDC85A	0.407	0.109	PECAM1	0.406	0.059
EMCN	0.304	0.051	PLXNA2	0.381	0.094
KIAA1549	0.204	0.032	PTPRB	0.475	0.074
NR3C2	0.53	0.237	ATP10A	0.828	0.152
TGM2	0.277	0.058	CNOT6L	0.421	0.136
AP3S1	0.35	0.107	PTPRM	0.764	0.333
FLI1	0.648	0.136	SLC16A1	0.334	0.054
LEF1	0.489	0.157	ELOVL7	0.811	0.194
ANGPT2	0.252	0.032	CMTM8	0.406	0.065
BACE2	0.376	0.107	SLC1A1	0.402	0.09
CGNL1	0.535	0.099	SLCO2B1	0.34	0.057
HERPUD1	0.263	0.061	RPGR	0.341	0.096
ITGA1	0.494	0.148	ANKS1A	0.502	0.172
PAM	0.512	0.2	CXorf36	0.303	0.067
PTPN14	0.341	0.097	DOCK9	0.779	0.326
RAPGEF4	0.491	0.175	SORBS2	0.558	0.164
SLC39A10	0.545	0.118	PDXK	0.334	0.085
TMEM132C	0.466	0.118	INPP5D	0.346	0.046
ACER2	0.274	0.046	MEF2A	0.61	0.269
ANO2	0.652	0.125	ABCB1	0.826	0.156
EPHA4	0.345	0.071	LMBR1	0.465	0.151
ERG	0.529	0.093	NEDD9	0.365	0.078
NOTCH4	0.237	0.034	SCARB1	0.431	0.125
RNF144B	0.388	0.084	SYNE1	0.713	0.287
SGPP2	0.381	0.054	THSD4	0.637	0.132
ST6GALNAC3	0.952	0.402	TFRC	0.338	0.057
USP6NL	0.404	0.114	PARVB	0.331	0.081
ABCG2	0.434	0.057	FLT1	0.931	0.22
ARHGAP31	0.399	0.133	IGF1R	0.712	0.323
IRAK3	0.434	0.098	SEC14L1	0.555	0.156
PODXL	0.437	0.067	ST6GAL1	0.472	0.129
PPFIBP1	0.671	0.22	RAPGEF1	0.469	0.168
PRICKLE2	0.442	0.173	WWTR1	0.539	0.201
RAPGEF2	0.648	0.307	NXN	0.569	0.171
RIMKLB	0.373	0.129	GPCPD1	0.302	0.092
SLC7A5	0.666	0.099	AKAP12	0.296	0.106
TBC1D4	0.623	0.153	PLEKHG1	0.645	0.183
CCNY	0.716	0.33	ESYT2	0.645	0.234
CPNE8	0.487	0.156	RBMS2	0.389	0.119
CRIM1	0.558	0.22	INSR	0.509	0.248
PDE10A	0.488	0.143	PTPRG	0.924	0.51
PRKCH	0.6	0.151			
ST8SIA6	0.521	0.073			
MECOM	0.795	0.166			
MYO10	0.32	0.1			

Cell Type: BEC, Venous

Gene	pct.1	pct.2
PDZRN3	0.644	0.254
TGM2	0.368	0.068
TLL1	0.131	0.004
FLI1	0.773	0.167
LEPR	0.41	0.088
SNTG2	0.427	0.015
TGFBR2	0.466	0.113
ADAMTS6	0.343	0.034
BACE2	0.553	0.115
ANO2	0.741	0.16
CDH11	0.59	0.164
ERG	0.582	0.123
RIMS2	0.313	0.042
RNF144B	0.45	0.103
SLC2A3	0.394	0.098
KCTD8	0.662	0.172
TBC1D4	0.703	0.184
THSD7A	0.575	0.151
CRIM1	0.695	0.237
MCTP1	0.531	0.075
PDE10A	0.568	0.164
PON2	0.509	0.149
PRKCH	0.696	0.179
CMIP	0.606	0.146
MECOM	0.817	0.214
BNC2	0.362	0.067
CHSY1	0.52	0.146
PECAM1	0.452	0.082
RAMP3	0.325	0.02
ATP10A	0.79	0.208
CADPS2	0.695	0.203
MKL2	0.834	0.217
SLC16A1	0.37	0.073
ELOVL7	0.882	0.237
CNKSR3	0.437	0.108
LDLRAD3	0.689	0.271
RALGAPA2	0.661	0.184
SLC1A1	0.552	0.103
SORBS2	0.626	0.19
SLC26A2	0.321	0.034
ABCB1	0.886	0.204
SCARB1	0.497	0.144
THSD4	0.664	0.17
ABCC4	0.502	0.069
TSHZ2	0.885	0.097
IL6R	0.305	0.035
SLC19A3	0.29	0.037
AFF3	0.755	0.172
LDB2	0.52	0.166
IL4R	0.541	0.085
PDE4D	0.849	0.371
HIPK3	0.607	0.174
ESYT2	0.723	0.26
BMPR2	0.703	0.254

Supplementary File 1. Page 2

Cell Type: Ependymal					
**Gene**	**pct.1**	**pct.2**	**Gene**	**pct.1**	**pct.2**
AGBL1	0.576	0.012	PARD3B	0.784	0.325
AGBL4	0.892	0.077	STK33	0.677	0.068
C8orf34	0.736	0.032	C4orf47	0.319	0.012
CCDC30	0.724	0.057	DNAH7	0.783	0.047
CCDC40	0.369	0.013	RFX3	0.832	0.364
CDHR3	0.468	0.06	WDR66	0.418	0.014
COL8A1	0.314	0.03	PLEKHA7	0.534	0.091
CRB1	0.635	0.093	DNAH12	0.632	0.016
DNAH10	0.311	0.003	STRBP	0.521	0.06
DNAH2	0.294	0.002	RFX2	0.596	0.069
FHAD1	0.394	0.005	NME5	0.327	0.018
GMPR	0.338	0.027	TTLL9	0.304	0.024
GRIN2A	0.422	0.027	CSPP1	0.566	0.103
HYDIN	0.834	0.06	DPP6	0.795	0.154
IQGAP2	0.265	0.011	HSP90AA1	0.615	0.285
ITGB8	0.432	0.098	KIAA1217	0.438	0.063
KIAA0319	0.344	0.035	SYNE1	0.81	0.342
NEK10	0.539	0.014	ACOT11	0.372	0.056
SH3BGR	0.143	0.019	PLEKHA5	0.758	0.23
SHANK2	0.51	0.017	DPP10	0.936	0.265
ST8SIA1	0.387	0.067	EFCAB2	0.6	0.13
TLN2	0.572	0.12			
ARMC2	0.452	0.051			
ARMC3	0.723	0.016			
BAIAP3	0.26	0.016			
EML6	0.343	0.047			
EYA1	0.482	0.066			
FAM81B	0.212	0.001			
FGF14	0.581	0.081			
IQCH	0.391	0.016			
MYLK3	0.252	0.001			
SPATA17	0.6	0.01			
VWA3A	0.557	0.01			
WDR78	0.474	0.026			
ANXA1	0.203	0.016			
EFHC1	0.404	0.039			
FAM184A	0.438	0.05			
FBXL13	0.385	0.026			
FZD3	0.338	0.04			
GYG2	0.3	0.019			
KIF27	0.355	0.034			
ULK4	0.757	0.086			
WWC1	0.36	0.048			
ANKRD26	0.377	0.075			
KATNAL2	0.337	0.05			
KCNMA1	0.722	0.167			
PPIL6	0.305	0.011			
ROBO2	0.46	0.053			
TMEM67	0.461	0.02			
TSGA10	0.518	0.049			
MAPK10	0.857	0.314			
NWD1	0.294	0.025			
SHROOM3	0.347	0.054			
IFT88	0.447	0.083			

Cell Type: Macrophage/ Microglia					
**Gene**	**pct.1**	**pct.2**	**Gene**	**pct.1**	**pct.2**
ARHGAP15	0.588	0.013	IPCEF1	0.328	0.014
FAM49B	0.544	0.121	NFATC2	0.262	0.027
FMN1	0.42	0.019	PREX1	0.392	0.149
HCK	0.164	0.003	SH3KBP1	0.345	0.13
IQGAP2	0.108	0.012	SOCS6	0.181	0.028
LRRK1	0.246	0.005	ARHGAP24	0.807	0.076
MANBA	0.221	0.045	CACNA1A	0.188	0.039
NCK2	0.298	0.085	GRID2	0.287	0.198
OSBPL3	0.231	0.049	PADI2	0.217	0.042
REL	0.151	0.029	RNF149	0.233	0.08
SAMSN1	0.198	0.002	SH3RF3	0.458	0.078
SKAP2	0.367	0.046	SLC1A3	0.725	0.325
TMEM156	0.146	0.004	SRGN	0.184	0.075
CD74	0.2	0.019	TBC1D14	0.212	0.039
CSF3R	0.132	0.001	BNC2	0.373	0.08
GALNT2	0.287	0.105	ELMO1	0.802	0.375
KCNK13	0.165	0.002	RHBDF2	0.29	0.014
KCNQ3	0.537	0.053	C3	0.388	0.013
LYN	0.317	0.026	ITPR2	0.656	0.261
POU2F2	0.244	0.006	ZNF710	0.245	0.04
PRAM1	0.131	0.004	CHST11	0.667	0.134
RUNX1	0.55	0.028	DOCK11	0.262	0.042
SMAP2	0.438	0.04	FGD4	0.531	0.182
ST8SIA4	0.203	0.031	MYO1F	0.288	0.012
TPK1	0.225	0.071	SLCO2B1	0.355	0.091
DENND3	0.29	0.086	MAML3	0.555	0.156
GRAMD1B	0.182	0.037	RBM47	0.401	0.007
MGAT4A	0.401	0.097	FMNL3	0.273	0.097
NHSL1	0.315	0.157	SFMBT2	0.643	0.202
OXR1	0.428	0.145	ANKRD44	0.594	0.269
PIK3AP1	0.2	0.005	DIAPH2	0.462	0.12
PTPRE	0.361	0.072	INPP5D	0.417	0.081
RAB31	0.276	0.069	MEF2A	0.734	0.307
SLC2A5	0.141	0.001	PLXDC2	0.929	0.272
DOCK8	0.647	0.019	SPTLC2	0.269	0.098
FOXP2	0.274	0.082	EPB41L2	0.61	0.225
GAB2	0.446	0.182	DOCK4	0.88	0.439
IL13RA1	0.2	0.042	ETV6	0.481	0.215
JAZF1	0.495	0.153	ABCC4	0.342	0.093
KCNMA1	0.495	0.166	TAB2	0.342	0.146
LHFPL2	0.42	0.1	GRB2	0.268	0.068
MEF2C	0.582	0.222	ST6GAL1	0.57	0.168
MLXIPL	0.121	0.004	ATM	0.302	0.118
SLC9A9	0.558	0.194	PLCG2	0.182	0.032
CELF2	0.787	0.302	PTPRJ	0.453	0.135
HS3ST4	0.637	0.015	FOXN3	0.674	0.31
PIK3R5	0.234	0.002	CTSB	0.224	0.031
PRKAG2	0.232	0.075	MTHFD1L	0.342	0.08
RIN3	0.282	0.021	AKAP13	0.505	0.221
SRGAP1	0.41	0.135	ABR	0.528	0.144
SRGAP2	0.645	0.154	FRMD4A	0.799	0.17
APBB1IP	0.544	0.007	SSH2	0.747	0.315
CYFIP1	0.319	0.093	FKBP5	0.661	0.351
ENTPD1	0.249	0.07			

Cell Type: Meningeal Fibroblast					
**Gene**	**pct.1**	**pct.2**	**Gene**	**pct.1**	**pct.2**
AGBL1	0.259	0.017	AKAP13	0.605	0.226
C8orf34	0.46	0.038	FCHSD2	0.603	0.323
CACNA2D3	0.673	0.168	EXT1	0.388	0.168
CHRM3	0.325	0.016	AHCYL2	0.617	0.215
DAPK1	0.241	0.061	CACNB2	0.703	0.185
DNAH14	0.28	0.008			
FAM20A	0.304	0.01			
FAT3	0.25	0.064			
GPC6	0.825	0.109			
NHSL2	0.278	0.056			
PKP2	0.25	0.009			
PLA2R1	0.315	0.055			
PTPN13	0.446	0.156			
SEMA3C	0.276	0.032			
SLIT2	0.39	0.051			
STXBP6	0.341	0.158			
SULF1	0.208	0.013			
EYA1	0.481	0.069			
EZR	0.192	0.062			
FAM91A1	0.32	0.073			
GNAL	0.152	0.032			
JAM3	0.416	0.14			
LEPR	0.551	0.109			
SLC4A4	0.755	0.101			
SNTG2	0.299	0.043			
CHSY3	0.505	0.082			
PTPRE	0.276	0.079			
SH3BP5	0.208	0.035			
FOXP2	0.509	0.085			
KCNMA1	0.78	0.172			
SH3PXD2A	0.355	0.098			
EYA2	0.514	0.057			
MAST4	0.631	0.298			
PRKAG2	0.362	0.078			
UAP1	0.178	0.016			
WDR86	0.133	0.005			
CDON	0.227	0.01			
CLU	0.414	0.147			
MRC2	0.255	0.039			
TMOD1	0.493	0.045			
TRPM3	0.294	0.138			
BNC2	0.818	0.085			
SLC7A2	0.467	0.044			
TBC1D8	0.35	0.084			
FBXO32	0.278	0.075			
SIPA1L1	0.612	0.255			
GMDS	0.598	0.213			
SLC26A2	0.313	0.053			
MAN1C1	0.264	0.068			
KIAA1217	0.28	0.066			
THSD4	0.731	0.202			
FOXP1	0.848	0.512			
PHLDB2	0.652	0.105			
SNED1	0.528	0.129			

Cell Type: Neuron

Gene	pct.1	pct.2	Gene	pct.1	pct.2
AGBL4	0.467	0.078	RIMS2	0.534	0.05
CALN1	0.337	0.051	ROBO1	0.476	0.171
CDH12	0.239	0.008	ROBO2	0.647	0.045
CDH13	0.318	0.034	ST6GALNAC5	0.24	0.009
CHRM3	0.448	0.008	TIAM1	0.434	0.097
CNTNAP2	0.81	0.11	ADARB2	0.351	0.159
DGKI	0.325	0.052	NRXN1	0.73	0.167
EPHA6	0.422	0.04	NRXN3	0.841	0.216
FSTL5	0.322	0.022	THRB	0.406	0.083
GABRB3	0.29	0.016	PCDH7	0.355	0.083
GABRG3	0.387	0.005	CACNA1A	0.447	0.034
GRIA2	0.423	0.084	LRP1B	0.763	0.325
GRIN2A	0.364	0.024	PLCB1	0.724	0.285
GRIP1	0.653	0.049	SPTBN4	0.308	0.025
GRM1	0.167	0.006	KCND2	0.498	0.052
GRM5	0.479	0.021	PPM1E	0.318	0.047
IQCJ-SCHIP1	0.403	0.125	ANKRD30BL	0.278	0.009
KSR2	0.318	0.011	NEGR1	0.621	0.108
LRRC4C	0.559	0.137	PHACTR1	0.528	0.191
LRRTM3	0.32	0.044	ANKS1B	0.738	0.233
MDGA2	0.553	0.066	NRG3	0.844	0.268
MYO16	0.208	0.015	DLGAP1	0.789	0.106
NMNAT2	0.29	0.011	KIAA1217	0.429	0.059
NRG1	0.131	0.005	RYR2	0.428	0.062
PTPRO	0.209	0.006	LRRTM4	0.609	0.09
PTPRT	0.193	0.014	ERBB4	0.671	0.356
RBFOX3	0.185	0.002	RBFOX1	0.83	0.071
RIMS1	0.368	0.033	FAM155A	0.784	0.196
SHANK2	0.257	0.017	KCNIP4	0.55	0.066
SLC35F3	0.22	0.019	DNM1	0.237	0.015
SLIT2	0.298	0.047	FRMD4A	0.739	0.173
SORCS1	0.201	0.035			
XKR4	0.49	0.017			
ATP8A2	0.471	0.014			
ATRNL1	0.531	0.048			
CNTN5	0.534	0.022			
CSMD1	0.795	0.061			
DSCAM	0.383	0.056			
ERC2	0.417	0.039			
FGF14	0.785	0.072			
KCNQ3	0.421	0.057			
KIF26B	0.213	0.03			
OPCML	0.723	0.057			
PCLO	0.409	0.03			
RALYL	0.298	0.021			
SHISA9	0.291	0.014			
GRIK1	0.416	0.025			
MACROD2	0.578	0.123			
OXR1	0.535	0.144			
RGS7	0.562	0.067			
SRRM4	0.261	0.002			
DAB1	0.631	0.141			
KCNQ5	0.511	0.012			
NBEA	0.576	0.208			

Cell Type: Oligodendrocyte

Gene	pct.1	pct.2	Gene	pct.1	pct.2	Gene	pct.1	pct.2
ATP1B1	0.216	0.022	SYNJ2	0.371	0.043	PSEN1	0.356	0.1
BCAS1	0.406	0.028	GPRC5B	0.326	0.093	DOCK5	0.648	0.11
C12orf76	0.202	0.019	KIF13B	0.327	0.098	GAB1	0.627	0.268
COBL	0.424	0.071	MAP7	0.767	0.133	RNF220	0.842	0.203
DNM3	0.785	0.318	NINJ2	0.156	0.009	ANK2	0.704	0.276
FA2H	0.328	0.014	NLGN1	0.573	0.118	FRYL	0.701	0.34
FAM107B	0.43	0.052	NRXN3	0.574	0.118	CCDC88A	0.536	0.174
MEGF10	0.342	0.07	PTPRD	0.861	0.137	MAGI2	0.92	0.515
MIR181A1HG	0.376	0.119	QDPR	0.416	0.038	LPGAT1	0.403	0.093
MOG	0.31	0.002	TTLL7	0.705	0.116	TMTC2	0.787	0.222
NALCN	0.398	0.085	ANK3	0.701	0.076	FRMD5	0.825	0.15
NECAB1	0.184	0.011	DOCK3	0.423	0.145	CLASP2	0.678	0.27
NKAIN2	0.786	0.109	MAPT	0.347	0.091	PPP2R2B	0.827	0.294
SLC44A1	0.86	0.179	PDE4B	0.891	0.258	MYO1D	0.372	0.113
SLCO1A2	0.437	0.051	TMEM144	0.657	0.056	DIP2B	0.64	0.203
ST18	0.885	0.07	PHLPP1	0.805	0.31	CDK19	0.58	0.142
UGT8	0.428	0.018	PLA2G16	0.248	0.045	SIK3	0.776	0.45
ZNF365	0.251	0.025	ZEB2	0.698	0.249			
ZNF536	0.65	0.041	CNDP1	0.239	0.012			
CD22	0.321	0.011	CTNNA3	0.934	0.169			
CDK18	0.358	0.034	ELMO1	0.843	0.239			
DNAJC6	0.572	0.074	IL1RAPL1	0.972	0.208			
DPYD	0.642	0.278	MAN2A1	0.851	0.241			
HHIP	0.33	0.013	P2RX7	0.236	0.027			
NCKAP5	0.688	0.158	RTN4	0.604	0.277			
NEO1	0.388	0.128	YPEL2	0.3	0.077			
PEX5L	0.741	0.062	ARHGAP21	0.55	0.192			
SEMA4D	0.262	0.031	ATG4C	0.338	0.069			
SLC24A2	0.839	0.07	DLG2	0.911	0.41			
SPOCK1	0.71	0.092	NCAM2	0.837	0.249			
VRK2	0.345	0.096	PLCL1	0.766	0.256			
AK5	0.476	0.032	QKI	0.947	0.616			
ATP8A1	0.638	0.096	SCD5	0.44	0.141			
CLMN	0.523	0.178	AGTPBP1	0.437	0.137			
CREB5	0.528	0.075	CERCAM	0.397	0.037			
DSCAML1	0.419	0.057	COL4A5	0.391	0.081			
EDIL3	0.746	0.138	DOCK10	0.739	0.104			
GPM6B	0.624	0.178	RAB40B	0.201	0.023			
LAMP2	0.382	0.071	ANKS1B	0.563	0.142			
NCAM1	0.705	0.188	DLG1	0.662	0.285			
PCSK6	0.424	0.022	FRMD4B	0.635	0.074			
PLD1	0.378	0.041	ASPA	0.198	0.015			
SH3GL3	0.586	0.033	MAP4K4	0.67	0.333			
SLC22A15	0.23	0.044	MAP4K5	0.491	0.199			
SRCIN1	0.346	0.021	PIP4K2A	0.871	0.219			
DPYSL5	0.253	0.013	CADM2	0.837	0.302			
ENPP2	0.526	0.035	PXK	0.523	0.078			
GAB2	0.393	0.123	ZDHHC20	0.351	0.098			
HECW2	0.347	0.045	POLR2F	0.37	0.035			
KCNMB4	0.304	0.039	PRUNE2	0.676	0.062			
OSBP2	0.226	0.043	PLEKHH1	0.435	0.03			
PDE1A	0.407	0.062	FMNL2	0.79	0.328			
ROBO1	0.386	0.111	SLC5A11	0.358	0.018			
SHROOM4	0.402	0.101	FUT8	0.57	0.225			

Cell Type: OPC

Gene	pct.1	pct.2	Gene	pct.1	pct.2
BCAS1	0.453	0.111	TRIM9	0.611	0.119
CA10	0.73	0.004	ZFPM2	0.701	0.214
CDH13	0.451	0.03	ITGA8	0.29	0.022
CNTN1	0.709	0.185	PCDH7	0.656	0.075
DGKI	0.388	0.05	CACNA1A	0.498	0.032
ESRRG	0.421	0.092	GRID2	0.875	0.184
GRIA2	0.639	0.078	LRP1B	0.959	0.319
GRM5	0.58	0.017	KCND2	0.818	0.043
GSG1L	0.254	0.007	SCD5	0.647	0.203
LRRC4C	0.96	0.126	TNK2	0.306	0.033
LRRTM3	0.499	0.039	NEGR1	0.555	0.108
LUZP2	0.733	0.015	ANKS1B	0.716	0.232
MAP2	0.652	0.17	CHST11	0.721	0.133
MDGA2	0.844	0.058	DPP6	0.904	0.143
MYO16	0.244	0.014	OPHN1	0.698	0.17
NOL4	0.379	0.052	DGKG	0.672	0.087
PTPRT	0.368	0.01	XYLT1	0.734	0.058
PTPRZ1	0.805	0.095	DLGAP1	0.695	0.106
RIMS1	0.307	0.034	KIAA1217	0.46	0.058
RNF150	0.402	0.053	KAT2B	0.608	0.162
SEZ6L	0.574	0.012	LRRTM4	0.901	0.082
SLC2A13	0.376	0.068	ERBB4	0.88	0.35
SORCS1	0.397	0.029	RBFOX1	0.507	0.076
STK32A	0.377	0.017	FAM155A	0.838	0.193
XKR4	0.498	0.015	KCNIP4	0.936	0.055
ATRNL1	0.791	0.04	LSAMP	0.959	0.425
CSMD1	0.957	0.055	PDZD2	0.798	0.311
DNER	0.412	0.088	EPN2	0.693	0.221
DSCAM	0.967	0.041	KIF13A	0.669	0.28
ERC2	0.359	0.039			
FGF14	0.916	0.067			
GLCCI1	0.379	0.076			
KIF26B	0.369	0.026			
OPCML	0.941	0.05			
PID1	0.534	0.125			
RALYL	0.368	0.018			
SHISA9	0.372	0.011			
TNR	0.917	0.02			
TOX	0.46	0.098			
GALNT13	0.724	0.087			
GRIK1	0.657	0.018			
MACROD2	0.476	0.124			
RAB31	0.391	0.066			
RGS7	0.507	0.067			
TMEM132C	0.642	0.155			
DAB1	0.658	0.139			
KCNMA1	0.653	0.162			
RIMS2	0.399	0.052			
ZDHHC14	0.54	0.154			
ADARB2	0.685	0.15			
ATP13A4	0.384	0.073			
NLGN1	0.944	0.211			
NRXN1	0.951	0.16			
THRB	0.394	0.082			

Supplementary File 1. Page 4

Cell Type: Pericyte

Gene	pct.1	pct.2	Gene	pct.1	pct.2
DGKB	0.54	0.101	GRK5	0.393	0.103
DOK6	0.215	0.027	PTEN	0.673	0.211
ENPEP	0.195	0.014	NID1	0.416	0.048
MPPED2	0.336	0.055	CRISPLD2	0.242	0.053
PCBP3	0.559	0.06	ZFHX3	0.771	0.244
PDE7B	0.832	0.16	DLC1	0.981	0.395
SH3RF1	0.44	0.129	ARHGAP10	0.604	0.09
SLC12A2	0.472	0.17	RORA	0.929	0.489
SNRK	0.386	0.102	PRKG1	0.899	0.301
ABCC9	0.376	0.046	ATP1B3	0.333	0.105
COL4A1	0.307	0.076	PARD3	0.856	0.403
FRMD3	0.602	0.087	BTBD9	0.602	0.293
KIAA1109	0.556	0.24	GJC1	0.457	0.03
PID1	0.442	0.063	SLC20A2	0.481	0.172
PTPRK	0.888	0.52			
SNTB1	0.473	0.072			
ZIC1	0.434	0.102			
COL4A2	0.35	0.11			
CTDSPL	0.353	0.105			
GRM3	0.654	0.21			
PDGFRB	0.506	0.028			
SGIP1	0.808	0.265			
CACHD1	0.422	0.141			
CACNA1C	0.823	0.216			
COL4A3	0.345	0.022			
CYTH3	0.462	0.086			
DAB1	0.351	0.104			
DACH1	0.427	0.128			
FOXP2	0.277	0.042			
NOTCH3	0.43	0.049			
PLCE1	0.393	0.097			
ADARB2	0.37	0.114			
COL4A4	0.307	0.02			
DENND2A	0.348	0.101			
EDNRA	0.215	0.02			
PTH1R	0.647	0.082			
RHOJ	0.442	0.099			
COLEC12	0.619	0.097			
INPP4B	0.567	0.136			
PLXDC1	0.354	0.022			
PDE3A	0.55	0.123			
SLC38A11	0.617	0.038			
ZEB1	0.681	0.301			
EBF1	0.851	0.254			
ARHGAP6	0.473	0.076			
KALRN	0.571	0.19			
PIK3CD	0.171	0.015			
AXL	0.236	0.041			
CALD1	0.754	0.181			
ATP1A2	0.755	0.157			
CPM	0.58	0.118			
RGL3	0.225	0.037			
GPC5	0.837	0.236			
EPS8	0.855	0.251			

Perivascular Fibroblast

Gene	pct.1	pct.2
ABCA6	0.454	0.034
ANTXR2	0.332	0.039
FAM189A1	0.207	0.036
FAM20A	0.15	0.008
FLRT2	0.754	0.083
NTRK3	0.856	0.228
PDZRN3	0.644	0.273
PRRX1	0.368	0.092
TBX18	0.296	0.054
TNXB	0.307	0.069
ABCA10	0.581	0.031
ATRNL1	0.272	0.053
FBLN1	0.55	0.034
FHL2	0.27	0.007
FLVCR2	0.291	0.036
JAK2	0.309	0.112
PID1	0.556	0.126
TNFRSF19	0.17	0.015
USP53	0.553	0.114
ABCA9	0.71	0.057
SRPX2	0.243	0.015
TMEM132C	0.516	0.159
CDH11	0.505	0.187
FOXP2	0.406	0.079
LAMA4	0.451	0.105
CELF2	0.737	0.305
LTBP2	0.138	0.003
PRKAG2	0.274	0.074
SYNPO2	0.275	0.081
COLEC12	0.54	0.188
ITGA8	0.16	0.026
GHR	0.293	0.075
LAMC1	0.42	0.089
ZEB1	0.713	0.366
LAMB1	0.238	0.015
PRICKLE1	0.345	0.021
SLC7A11	0.45	0.173
TGFBR3	0.525	0.103
RBMS3	0.632	0.243
PIAS1	0.563	0.265
NID1	0.385	0.112
FNDC3B	0.547	0.258
CENPP	0.38	0.101
RORA	0.945	0.564
SVIL	0.558	0.176

Smooth Muscle Cell (SMC)

Gene	pct.1	pct.2
COL18A1	0.362	0.102
TBX18	0.282	0.047
COL4A1	0.419	0.104
DSTN	0.276	0.062
COL4A2	0.466	0.139
DYNC1I1	0.311	0.111
CACNA1C	0.883	0.302
KCNAB1	0.527	0.088
MSRB3	0.321	0.115
NOTCH3	0.442	0.104
PLCE1	0.431	0.138
FRY	0.467	0.205
SYNPO2	0.286	0.074
INPP4B	0.552	0.199
ITGA8	0.19	0.02
PIP5K1B	0.493	0.083
ADCY3	0.256	0.05
LTBP1	0.432	0.118
PDE3A	0.722	0.176
SH3RF3	0.344	0.073
SLC38A11	0.522	0.128
EBF1	0.833	0.342
ARHGAP6	0.413	0.137
NTN4	0.165	0.025
DMD	0.573	0.289
SETBP1	0.449	0.143
CALD1	0.729	0.265
FAM129A	0.247	0.026
RGS6	0.683	0.105
SLIT3	0.697	0.033
SORBS2	0.51	0.204
EPS8	0.771	0.344
RYR2	0.249	0.06
LMOD1	0.219	0.012
MYL9	0.397	0.051
TBC1D1	0.427	0.16
PHLDB2	0.315	0.095
PDLIM5	0.459	0.215
CRISPLD2	0.424	0.071
ZFHX3	0.884	0.315
ARHGAP10	0.587	0.165
PRKG1	0.933	0.387
RBPMS	0.696	0.239
CACNB2	0.548	0.167
LPP	0.822	0.45
MYH11	0.36	0.013

T Cell

Gene	pct.1	pct.2	Gene	pct.1	pct.2
ARHGAP15	0.63	0.025	DOCK11	0.236	0.047
EMB	0.322	0.004	MYO1F	0.288	0.017
ERN1	0.16	0.022	BCL2	0.484	0.266
FAM49B	0.396	0.13	FAM129A	0.234	0.037
IKZF3	0.234	0.001	RNF213	0.256	0.093
IQGAP2	0.444	0.013	ANKRD44	0.698	0.275
MDFIC	0.302	0.042	MBNL1	0.855	0.451
PRKCQ	0.311	0.026	ZC3HAV1	0.245	0.071
SAMSN1	0.157	0.007	EML4	0.362	0.08
SLC38A1	0.319	0.054	EPB41	0.276	0.052
SLFN12L	0.433	0.028	PDE7A	0.427	0.088
STK17B	0.205	0.012	STIM1	0.291	0.088
TTC39C	0.197	0.018	ATM	0.322	0.122
CLEC2D	0.288	0.029	STK10	0.256	0.04
GLCCI1	0.219	0.083	RASSF3	0.225	0.06
KCNAB2	0.134	0.008	CCND3	0.567	0.137
KLRD1	0.225	0.028	RABGAP1L	0.681	0.326
RASA3	0.214	0.034			
RUNX1	0.419	0.039			
SLC16A7	0.199	0.041			
SMAP2	0.256	0.049			
SPON2	0.154	0.002			
TOX	0.413	0.106			
TNFAIP8	0.362	0.064			
CARD11	0.311	0.004			
CD247	0.501	0.006			
DOCK8	0.402	0.033			
KCNQ5	0.16	0.021			
LITAF	0.165	0.041			
PRKCB	0.447	0.068			
PTPN22	0.188	0.002			
STK17A	0.188	0.019			
TRERF1	0.251	0.038			
CBLB	0.684	0.215			
CELF2	0.761	0.313			
INPP4A	0.279	0.098			
PIK3R5	0.197	0.007			
TC2N	0.219	0.005			
APBB1IP	0.271	0.019			
CCDC88C	0.199	0.018			
IPCEF1	0.145	0.021			
NFATC2	0.231	0.032			
PRKCH	0.661	0.213			
SYTL3	0.439	0.008			
AKNA	0.268	0.023			
SIDT1	0.168	0.013			
SKAP1	0.664	0.002			
STK4	0.333	0.067			
DIAPH1	0.248	0.051			
FYN	0.701	0.349			
CNOT6L	0.516	0.176			
RASA2	0.416	0.168			
CDC42SE2	0.43	0.111			
CHST11	0.593	0.145			
