# Supplementary material for: Reduced RNA adenosine-to-inosine editing in hippocampus vasculature associated with Alzheimer’s disease
Source: Brain Commun. 2022 Sep 22;4(5):fcac238. doi: 10.1093/braincomms/fcac238 (PMC9527665; doi:10.1093/braincomms/fcac238)
Supplement: fcac238_Supplementary_Data [file fcac238_supplementary_data.zip › Supplementary_figures.pdf]

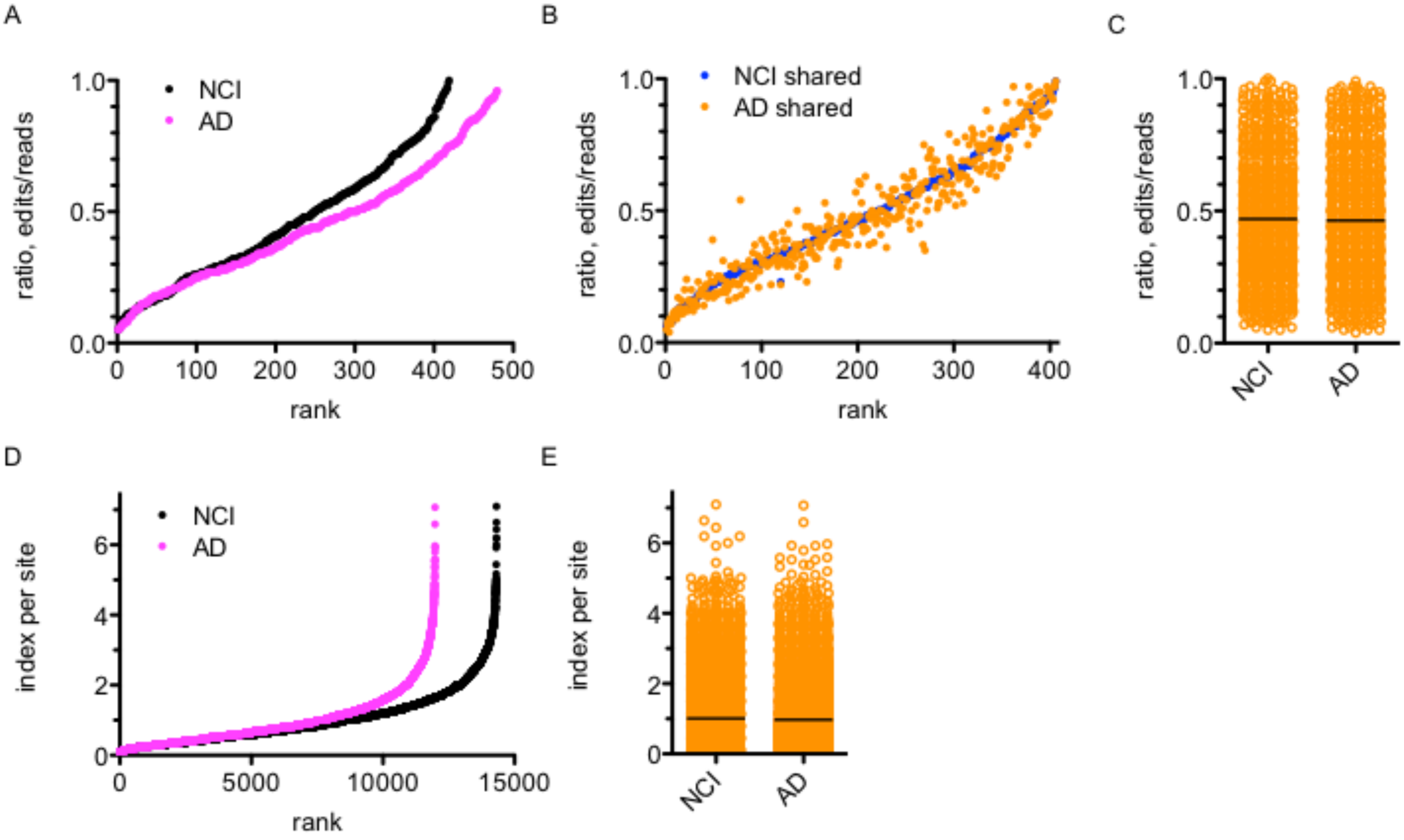

Supplementary Figure 1. Comparison of endogenous Alu RNA A-to-I editing between NCI and AD in dorsolateral prefrontal cortex. (A) Number of A-to-I editing sites present in either all samples within the NCI cohort or all samples within the AD cohort. Y-axis is ratio of edits/reads at each edited site, X-axis is rank. P>0.05 $\chi 2$ analysis. (B) Number of editing sites shared between all samples in both cohorts. Y-axis is ratio of edits/reads at each edited site, X-axis is rank. (C) As in (A&B) except all editing sites are presented as a dot plot; black line is the mean. P>0.05, t test with Welch's correction. (D) NCI and AD genome-wide editing indices. Index at each site was determined by multiplying the edit/read ratio by the number of samples in each cohort with edits, X-axis; Y-axis is the rank. P>0.05 $\chi 2$ analysis (E) As in (F) except results are presented as a dot plot; black line is the mean. P>0.05, t test with Welch's correction

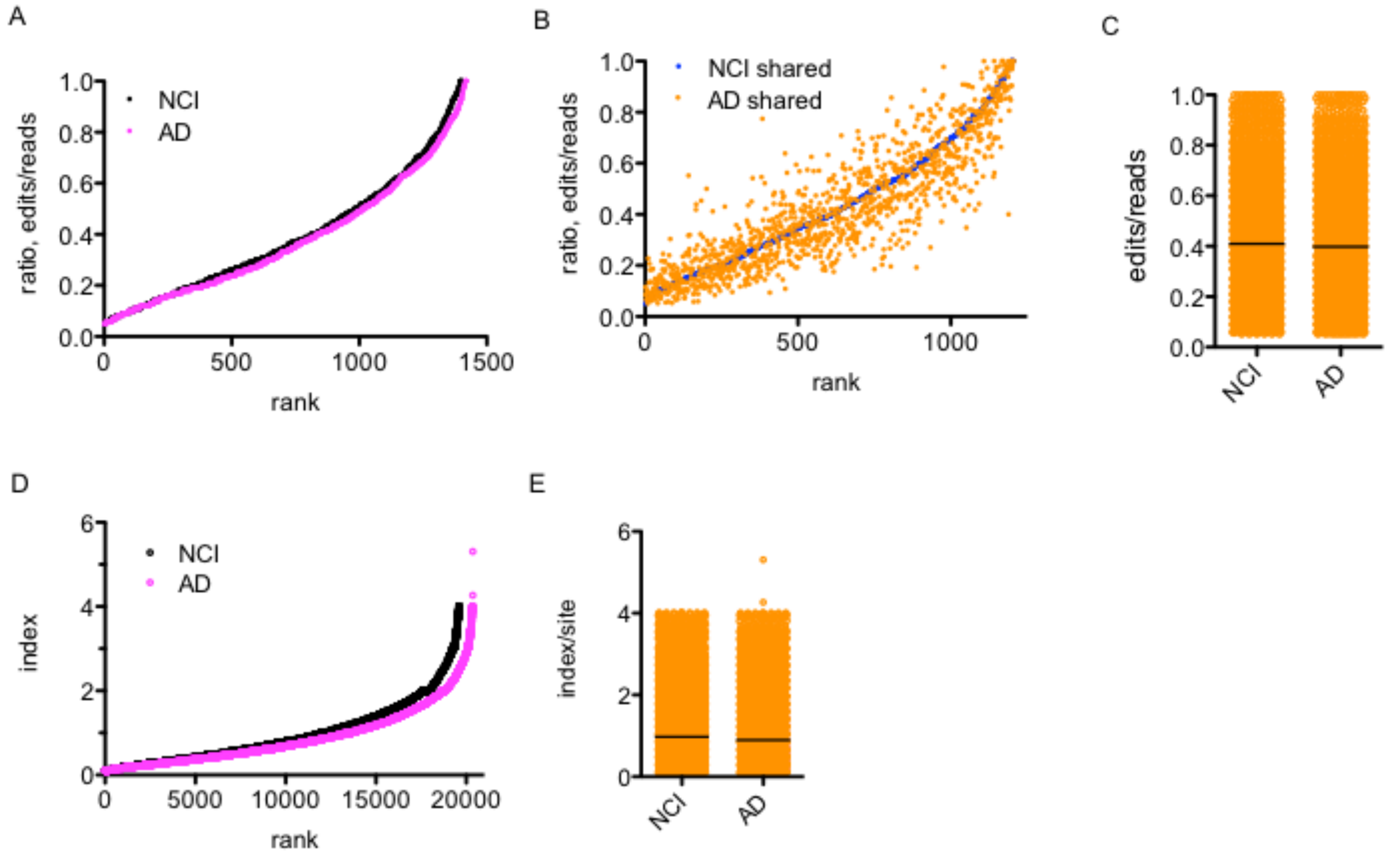

Supplementary Figure 2. Comparison of A-to-I editing in prefrontal cortex vasculature between NCI and AD cohorts. (A) Number of A-to-I editing sites present in all samples within the NCI cohort or all samples within the AD cohort. Y-axis is ratio of edits/reads at each edited site, X-axis is rank, P>0.05, χ^2 analysis comparing number of editing sites. (B) Number of A-to-I editing sites shared between all samples within both NCI and AD cohorts. Y-axis is ratio of edits/reads at each edited site, X-axis is rank, (C) As in (A&B) except results are presented as a dot plot; black line is the mean, P>0.05, t-test with Welch's correction. (D) NCI and AD genome-wide editing indices. Index at each site was determined by multiplying the edit/read ratio by the number of samples in each cohort with edits, X-axis; Y-axis is the rank, P>0.05, χ^2 analysis comparing number of editing sites (E) As in (D) except results are presented as a dot plot; black line is the mean, P>0.05, t-test with Welch's correction.

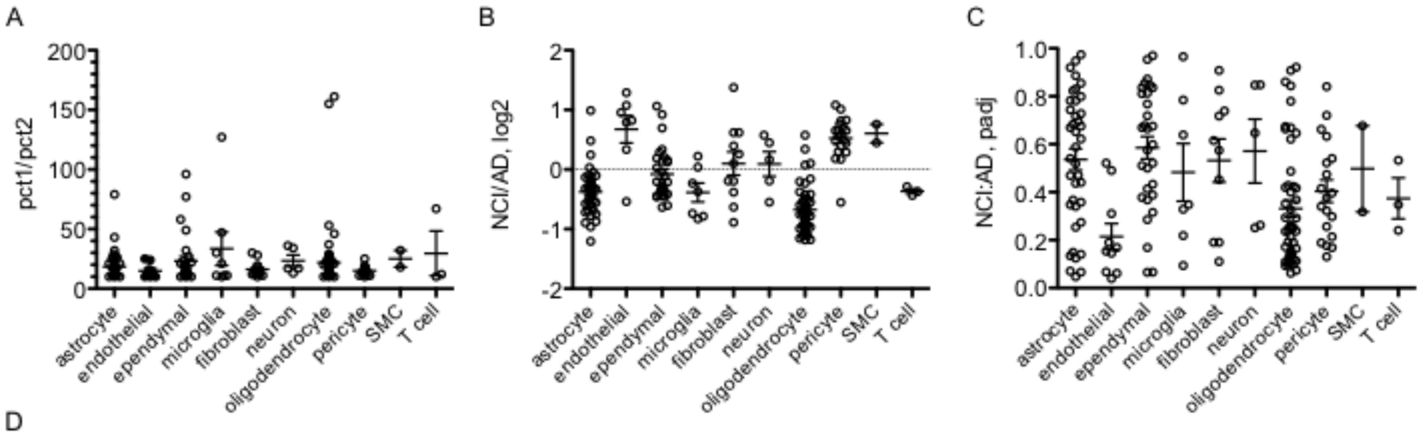

D

Gene, log2FoldDifference, Wald statistic, BH padj

__astrocyte__: ***ACOT11***, -0.10, -0.23, 0.92; ***ADCY2***, -0.75, -1.83, 0.27; ***ADGRA3***, -0.70, -2.32, 0.15; ***ADGRV1*** ,-0.34, -0.76, 0.70; ***AQP4***, -0.14, -0.46, 0.83; ***AQP4.AS1***, -1.20, -2.45, 0.12, ***ATP13A4***, -0.72, -1.55, 0.37; ***BMPR1B***, -0.96, -2.36, 0.14; ***C1orf61***, -0.90, -2.40, 0.13; ***CD44***, 0.48,, 0.87, 0.65; ***COL21A1***, -1.00, -2.82, 0.07; ***DAPK1***, -0.40, -0.93, 0.62; ***DNAH7***, -0.64,, -2.85, 0.07; ***EFEMP1***, -0.18, -0.48, 0.82; ***FAM189A2***, 0.24, 0.81, 0.68; ***FAT3***, -0.33, -0.72, 0.72; ***FGFR3***, -1.10, -3.00, 0.05; ***GABRB1***, -0.55, -1.35, 0.44; ***GFAP***, -0.87, -1.90, 0.25; ***IQCA1***, -0.21, -0.39, 0.85; ***LGI1***, -0.57, -1.62, 0.34; ***LINC01094***, -0.70, -1.28, 0.47; ***MAOB***, -0.27, -0.54, 0.80; ***MAP3K4***, -0.13, -0.57, 0.78; ***MED12L***, -0.29, -1.28, 0.47; ***MGST1***, -0.40, -0.80, 0.68; ***MRVI1***; -0.22, -0.57, 0.78; ***NPL***, -1.26, -3.05, 0.05; ***NRCAM***, -0.47, -1.11, 0.54; ***PTPRZ1***, 0.03, 0.08, 0.97; ***RFX4***, -0.31, -0.67, 0.74; ***RGMA***, 0.04, -0.15, 0.95; ***RORB***, -0.58, -1.17, 0.52; ***SLC14A1***, -0.77, -1.57, 0.36; ***SLC16A9***, 0.54, -1.60, 0.35; ***SLC1A2***, -0.32, -0.70, 0.73; ***SLC25A18***, -0.45, -1.01, 0.59; ***SLC4A4***, -0.53, -1.36, 0.44; ***TPD52L1***, -0.10, -0.32, 0.89; ***WDR49***, -0.32, -0.83, 0.67;

__endothelial:__ ***BTNL9***, 1.14, 2.91, 0.06; ***GPR85***, -0.54, -1.16, 0.52; ***ACKR1***, 0.95, 2.24, 0.17; ***ADAMTS6***, 0.33, 2.34, 0.15; ***ADGRG6***, 0.79, 2.31, 0.15; ***FENDRR***, 1.29, 2.84, 0.07; ***PLA1A***, 1.08, 2.14, 0.19; ***RAMP3***, 0.82, 1.72, 0.31;

__ependymal__: ***CCDC30***, -0.04, -0.14, 0.95; ***CEP126***, 0.15, 0.51, 0.81; ***CFAP43***, -0.39, -1.16, 0.52; ***CFAP44***, 0.03, 0.09, 0.97; ***CFAP46***, -0.23, -0.81, 0.68; ***CFAP54***, -0.46, -1.49, 0.39; ***CFAP70***, -0.34, -0.98, 0.60; ***COL8A1***, 1.06, 2.24, 0.17; ***DNAH11***, 0.92, 2.85, 0.07; ***DNAH7***, -0.64, -2.85, 0.07; ***EFHC1***, -0.35, -1.70, 0.32; ***FAM227A***, -0.29, -0.84, 0.66; ***FBLN5***, 0.69, 1.79, 0.29; ***GMPR***, -0.46, -1.42, 0.41; ***HYDIN***, 0.29, 0.81, 0.68; ***IQCA1***, -0.21, -0.39, 0.85; ***IQCG***, 0.12, 0.45, 0.84; ***KIF27***, 0.33, 0.72 ,0.72; ***LRRIQ1***, -0.45, -1.18, 0.51; ***MAATS1***, 0.14, 0.44, 0.84; ***MOK***, -0.21, -0.61, 0.77; ***NPHP1***, -0.41, -1.54, 0.37; ***PPARGC1A***, 0.19, 0.42, 0.85; ***SPAG8***, -0.61, -1.51, 0.38; ***SPEF2***, 0.20, 0.50, 0.81; ***TMEM67***, -0.12, -0.35, 0.87; ***TSGA10***, -0.45, -1.37, 0.43; ***WDR49***, -0.32, -0.83, 0.67; ***ZNF273***, -0.41, -1.04, 0.58;

__microglia__: ***C3***, -0.79 -2.03. 0.22; ***CD74***, 0.22, 0.57, 0.78; ***MS4A6A***, -0.83, -1.66, 0.33; ***PTPRC***, -0.37, -1.60, 0.35; ***RHBDF2***, 0.04, 0.11, 0.97; ***SLC8A1***, -0.74, -2.64, 0.09; ***SMAP2***, -0.23, -0.90, 0.64;

__fibroblast__: ***BNC2***, 0.26, 0.7, 0.7; ***COL11A1***, 0.1, 0.27, 0.9; ***FAM20A***, 0.62, 2.14, 0.19; ***IGFBP6***, -0.63, -1.3, 0.45; ***SLC7A2***, -0.19, -0.5, 0.8

__neuron__: ***CACNA1A***, -0.55, -1.9, 0.25; ***CACNA1E***, 0.17, 0.4, 0.85; ***CADPS***, -0.2, -0.4, 0.85; ***GRIK1***, 0.58, 1.9, 0.26; ***SYT1***, 0.45, 0.88, 0.65;

__oligodendrocyte__: ***AATK***, -1.06, -2.55, 0.11; ***AMER2***, -1.11, -2.48, 0.12; ***ASPA***, -0.99, -2.36, 0.14; ***ATP10B***, -1.36, -2.78, 0.07; ***ATP1B1***, -0.74, -2.13, 0.19; ***BCAS1***, -0.94,-2.23, 0.17; ***C10orf90***, -0.23, -0.43, 0.84; ***C12orf76***, -0.97, -2.30, 0.16; ***CD22***, -1.33, -3.00, 0.05; ***CDH19***, -0.86, -1.76, 0.29; ***CDK18***, -0.60, -1.46, 0.40; ***CERCAM***, -1.36, -3.06, 0.05; ***CNDP1***, -0.65, -1.41, 0.42; ***CNP***, -0.55, -1.59, 0.35; ***CNTN2***, -1.08, -2.88, 0.06; ***ENPP2***, -0.74, -1.95, 0.24; ***GPR37***, -0.89, -2.05, 0.21; ***HS3ST5***, -1.48, -2.54, 0.11; ***KCNH8***, -0.48, -0.820.67; ***LRP2***, -1.23, -2.52, 0.11; ***MAL***, -0.78, -2.26, 0.16; ***MOG***, -0.80, -1.67, 0.33; ***MYRF***, -0.99, -2.52, 0.11; ***NECAB1***, -0.73, -2.40, 0.13; ***PALM2***, -0.93, -1.91, 0.25; ***PCSK6***, -0.81, -1.76, 0.29; ***PIEZO2***, -0.92, -2.15, 0.19; ***POLR2F***, -0.65, -1.89, 0.26; ***PRUNE2***, -0.66, -2.39, 0.13; ***QDPR***, -0.51, -1.39, 0.42; ***SCD***, -0.37, -0.93, 0.62; ***SH3GL3***, -1.01, -2.32, 0.15; ***SH3TC2***, -0.76, -1.96, 0.24; ***SHTN1***, -1.18, -2.79, 0.07; ***ST18***, -1.08, -2.35, 0.14; ***TF***, -0.68, -1.54, 0.37; ***TMEM144***, -0.94, -1.96, 0.24; ***UGT8***, -1.18, -2.89, 0.06; ***ZNF536***, -1.15, -2.65, 0.09; ***ARPP21***, -0.41, -0.83, 0.67; ***CACNA1A***, -0.55, -1.91, 0.25; ***COL11A1***, 0.10, 0.27, 0.91; ***GRIA3***, -0.20, -0.58, 0.78; ***GRIK1***, 0.58, 1.87, 0.26; ***KCNIP4***, -0.70, -2.61, 0.10; ***LRRTM3***, -1.11, -2.60, 0.10; ***MMP16***, -0.16, -0.38, 0.86; ***PCDH15***, -0.89, -1.91, 0.25; ***SCN3A***, 0.34, 0.85, 0.66; ***SMOC1***, -0.60, -1.40, 0.42; ***TNR***, -0.42, -0.88, 0.65; ***UNC80***, 0.07, 0.23, 0.92; ***VCAN***, -0.51, -1.23, 0.49; ***XYLT1***, -0.65, -1.38, 0.43; ***ABCA10***, -0.38, -1.04, 0.57; ***ABCA6***, -0.89, -2.54, 0.11; ***ABCA9***, -0.19, -0.68, 0.74; ***FAM20A***, 0.62, 2.14, 0.19; ***FBLN1***, 0.39, 0.95, 0.62;

__pericyte__: ***COL4A3***, 0.65, 2.05, 0.21; ***EDNRA***, 0.83, 2.15, 0.19; ***ENPEP***, 1.01, 1.68, 0.32; ***GJC1***, 0.29, 0.91, 0.63; ***LAMA2***, 0.56, 1.89, 0.26; ***LAMC3***, 0.50, 1.42, 0.42; ***LZTS1***, 1.08, 2.87, 0.06; ***NDUFA4L2***, 0.82, 2.42, 0.13; ***P2RY14***, 0.18, 0.71, 0.72; ***PDGFRB***, 0.66, 2.24, 0.17; ***PIK3CD***, -0.55, -1.47, 0.40; ***PLXDC1***, 0.39, 1.26, 0.47; ***RGS5***, 0.70, 1.75, 0.30; ***SLC12A7***, 0.44, 1.16, 0.52; ***SLC19A1***, 0.17, 0.44, 0.84; ***SLC30A10***, 0.76, 2.20, 0.18; ***SLC38A11***, 0.30, 0.85, 0.66; ***SLC6A12***, 0.59, 1.52, 0.38; ***SLC6A13***, 0.65, 1.63, 0.34; ***SMOC2***, 0.48, 1.1, 0.54

__SMC__: ***LMOD1***, 0.76, 1.69, 0.32; ***MYOCD***, 0.45, 0.81, 0.68;

__T cell__: ***AKNA***, -0.29, -1.13, 0.53; ***PTPRC***, -0.37, -1.60, 0.35; ***STK17A***, -0.44, -1.95, 0.24

Supplementary Figure 3. Expression levels of lineage specific genes in HPC vasculature are similar between NCI and AD cohorts. (A) Y-axis is the pct1/pct2 ratio, pct1 is the percentage of cells in a cluster where the expressed gene is detected, pct2 is the percentage of cells in all other clusters where the gene is detected. Symbols show genes with pct1/pct2 ratios >10 for each lineage. (B) Average NCI/AD expression ratios, log_2, for each gene identified in (A), symbols represent individual genes; Number of samples in each group; NCI: # = 7, AD: # = 7. (C) Benjamini Hochberg adjusted p values (Y-axis) comparing expression levels of each gene shown in (A) between NCI and AD cohorts shown in (B). (D) List of genes with pct1/pct2 ratios >10 in indicated cell lineages. Number of genes used in analysis of each lineage: astrocytes, #=39; endothelial, #=10; ependymal, #=30; microglia, #=7; fibroblast, #=10; neuron, #=5; oligodendrocyte, #=50; pericyte, #=19; smooth muscle cell (SMC); #=2; T cell, #=3. Following each gene symbol (bold) is the NCI:AD log2FoldDifference, Wald statistic and the Benjamini Hochberg adjusted p value (DESeq2).
