# Supplementary material for: Reduced RNA adenosine-to-inosine editing in hippocampus vasculature associated with Alzheimer’s disease
Source: Brain Commun. 2022 Sep 22;4(5):fcac238. doi: 10.1093/braincomms/fcac238 (PMC9527665; doi:10.1093/braincomms/fcac238)
Supplement: fcac238_Supplementary_Data [file fcac238_supplementary_data.zip › SupTable1.docx]

**Supplementary Table 1**. DNA sequence of unedited and edited MDM4 AluSg4

>hg19 build, AluSg4 range=chr1:204505114-204505394

unedited

GTGCGGTGGCTCATGCCTGT**A**ATCCCAGCACTTTGGG**A**GGCCG**A**GGCGGG

TGGATCAC**AA**AGTCAGGAGTTTGAGACCAGCCTGGCC**A**AGATGAT**A**AAAT

CCTGTCTCTACTAAAAATACAAAAATTAGTCAGGCGTGCGCCTAT**A**ATCC

TAGCTACTTGGGAGGCTGAGGCAGGAGAATCACTTGAGCCAGGGAGGTAG

AGGTTTTAGTGAGCCGAGATTGCGCCACTGCTCTCCAGCCAGGGTGACAG

AGCAAG**A**CTCCGTCTCAAACAAACAAAAAAA

edited

GTGCGGTGGCTCATGCCTGT**G**ATCCCAGCACTTTGGG**G**GGCCG**G**GGCGGG

TGGATCAC**GG**AGTCAGGAGTTTGAGACCAGCCTGGCC**G**AGATGAT**G**AAAT

CCTGTCTCTACTAAAAATACAAAAATTAGTCAGGCGTGCGCCTAT**G**ATCC

TAGCTACTTGGGAGGCTGAGGCAGGAGAATCACTTGAGCCAGGGAGGTAG

AGGTTTTAGTGAGCCGAGATTGCGCCACTGCTCTCCAGCCAGGGTGACAG

AGCAAG**G**CTCCGTCTCAAACAAACAAAAAAA

A nucleotides (unedited) changed to G nucleotides (edited) are identified by boldface.
